# Molecular Characterization of Highly Pathogenic Avian Influenza Viruses H5N6 Detected in Denmark in 2018–2019

**DOI:** 10.3390/v13061052

**Published:** 2021-06-02

**Authors:** Yuan Liang, Jesper Schak Krog, Pia Ryt-Hansen, Anders Gorm Pedersen, Lise Kirstine Kvisgaard, Elisabeth Holm, Pernille Dahl Nielsen, Anne Sofie Hammer, Jesper Johannes Madsen, Kasper Thorup, Lars Erik Larsen, Charlotte Kristiane Hjulsager

**Affiliations:** 1 Department of Veterinary and Animal Sciences, University of Copenhagen, 1870 Frederiksberg, Denmark; yuan.liang@sund.ku.dk (Y.L.); piarh@sund.ku.dk (P.R.-H.); likik@sund.ku.dk (L.K.K.); hammer@sund.ku.dk (A.S.H.); lael@sund.ku.dk (L.E.L.); 2 Department of Virus and Microbiological Special Diagnostics, Statens Serum Institut, 2300 Copenhagen S, Denmark; jskr@ssi.dk (J.S.K.); elho@ssi.dk (E.H.); 3 DTU Health Tech, Bioinformatics, Technical University of Denmark, 2800 Kgs. Lyngby, Denmark; agpe@dtu.dk; 4 Animal Health Division, Danish Veterinary and Food Administration, 2600 Glostrup, Denmark; perni@fvst.dk; 5 Natural History Museum of Denmark, University of Copenhagen, 1350 Copenhagen, Denmark; jjmadsen@snm.ku.dk; 6 GLOBE Institute, University of Copenhagen, 1350 Copenhagen, Denmark; kasper.thorup@sund.ku.dk

**Keywords:** influenza A virus, H5N6 subtype, highly pathogenic avian influenza, birds, wild, Europa, disease outbreaks, phylogeny, surveillance, infectious disease outbreak

## Abstract

Beginning in late 2017, highly pathogenic avian influenza (HPAI) H5N6 viruses caused outbreaks in wild birds and poultry in several European countries. H5N6 viruses were detected in 43 wild birds found dead throughout Denmark. Most of the Danish virus-positive dead birds were found in the period from February to April 2018. However, unlike the rest of Europe, sporadic HPAI H5N6-positive dead wild birds were detected in Denmark in July, August, September, and December 2018, with the last positive bird being found in January 2019. HPAI viruses were not detected in active surveillance of apparently healthy wild birds. In this study, we use full genome sequencing and phylogenetic analysis to investigate the wild bird HPAI H5N6 viruses found in Denmark. The Danish viruses were found to be closely related to those of contemporary HPAI H5N6 viruses detected in Europe. Their sequences formed two clusters indicating that at least two or more introductions of H5N6 into Denmark occurred. Notably, all viruses detected in the latter half of 2018 and in 2019 grouped into the same cluster. The H5N6 viruses appeared to have been maintained undetected in the autumn 2018.

## 1. Introduction

Highly pathogenic avian influenza (HPAI) H5N1 virus (A/goose/Guangdong/1/96) was first detected in China [1] and has since then evolved into clades 0–9 and multiple subclades [2]. Clade 2.3.4.4 viruses have since 2014 been introduced into Europe on several occasions, where they have caused multiple outbreaks in wild birds and poultry [3,4,5,6,7,8,9]. The periodic incursions likely occurred through wild bird migration [10,11,12]. The general consensus is that HPAI H5 clade 2.3.4.4 viruses spread via the migration of wild birds from Asia to the border between Russia and Mongolia with subsequent migration and dissemination of viruses into Europe [3]. Clade 2.3.4.4b H5N8 viruses have especially caused numerous outbreaks in wild birds and substantial losses in domestic poultry in 2016–2017 [13,14,15]. From late 2017 and continuing into 2018, outbreaks in wild birds and poultry caused by HPAI H5N6 clade 2.3.4.4b viruses were reported in several European countries [16]. These viruses were reassortants of H5N8 clade 2.3.4.4b from 2016–2017 that contained polymerase basic protein 2 (PB2) and neuraminidase (NA) gene segments from European low pathogenic avian influenza (LPAI) viruses [4]. HPAI H5N6 virus was also detected in early 2017 in a backyard poultry flock in Greece. However, phylogenetic analyses have shown that these viruses differed genetically from the other European HPAI H5N6 viruses by containing an N6 most closely related to contemporary Asian HPAI H5N6 viruses, while all the remaining segments were most closely related to the 2016–2017 European H5N8 viruses. Thus, the Greek viruses were not directly ancestral to the H5N6 viruses found in European countries later in 2017 and through to 2019 [4,17].

Seven outbreaks with HPAI H5N6 in poultry and captive birds were reported through the Animal Disease Notification System (ADNS) from the Netherlands, Germany, and Sweden in the period from December 2017 to March 2018. In addition, an outbreak in captive birds was reported from Germany in August 2018. Wild bird detections were reported through ADNS from the Netherlands, Switzerland, Germany, the United Kingdom, Sweden, Ireland, Denmark, Finland, and Slovakia until May 2018. Later detections were reported in a single wild bird in the United Kingdom in mid-June and in two wild birds in the Netherlands in late August. In Denmark, HPAI H5N6 viruses were detected in wild birds from February 2018 until January 2019.

Several pheasants infected with HPAI H5N6 virus were found in August–September 2018. In Denmark, approximately 1,000,000 pheasants are released each year between 1 April and 31 August for restocking [18]. Pheasants have previously been found to become persistently infected with the LPAI virus [19,20], which prompted the question of whether pheasants could be potential silent reservoirs for the maintenance of HPAI viruses and were the reason for H5N6 virus detections in wild birds later in 2018 than in any other European country.

AIV with the H5N6 subtype has caused human cases in China since 2014. As of 28 April 2021, a total of 31 laboratory-confirmed cases of human infection with H5N6 virus, including eight deaths, have been reported to the WHO [21]. However, these viruses differ genetically from the H5N6 viruses detected in avian species in Europe. Even so, it is important to elucidate the mechanisms and risk factors for increased infectivity, virulence, and the potential for crossing the species barrier of the HPAI H5N6 viruses found in Europe.

The aim of this study was to characterize the HPAI H5N6 viruses detected in Denmark by full genome sequencing and phylogenetic analyses to investigate if the outbreaks were due to single or multiple incursions of the virus. Furthermore, since HPAI H5N6-positive wild pheasants were detected during the late phase of the outbreak, a cross-sectional study was performed to investigate potential role of pheasants as a reservoir of HPAI H5N6 viruses.

## 2. Materials and Methods

### 2.1. Sampling

For avian influenza virus (AIV) active surveillance, apparently healthy wild birds were sampled in connection with bird ringing or hunting activities in 2018–2019, mainly from September to December. Mainly species belonging to the Anatidae family were sampled, based on the experience that these most often are infected with AIV. One cloacal swab was obtained from each bird and tested for the presence of AIV by PCR in pools of up to five swabs from birds of the same species, sampled at the same time and location.

Wild birds found dead in the environment were tested as part of the EU mandatory passive surveillance for AIV throughout 2018–2019. Each bird was tested for the presence of AIV in a pool of cloacal and tracheal swabs. The sampled birds included mainly species considered to be AIV high-risk target species [22]. During the outbreak, when HPAI virus-positive birds were found at a given geographical location, testing was suspended for the following month in a zone of 20 km from the positive finding.

For the cross-sectional study of subclinical HPAI in wild pheasants, clinically healthy wild pheasants were captured for sampling at five different locations in the region surrounding the Danish waters “Smaalandsfarvandet” south of Zealand during January to March 2019. A blood sample and a cloacal swab sample was obtained from each bird, and the birds were subsequently released.

### 2.2. Virus Detection, Subtyping and Antibody Detection

Viral RNA was extracted using the RNeasy Mini Kit (QIAGEN, Copenhagen, Denmark). Samples were tested with PCR assays recommended by the European Reference Laboratory for Avian Influenza (EURL). Initially, samples were tested for AIV by M-gene real-time RT-PCR [23] and subtyped for H5 [24,25] or H7 [26] by real-time RT-PCR. Subtype confirmation and pathogenicity determination was performed by sequencing across the hemagglutinin (HA) cleavage site (CS) using the PCR fragment from the KHA H5 CS conventional RT-PCR assay [27] or H5 CS real-time RT-PCR assays [24]. Serum was separated from the blood samples collected for the cross-sectional pheasant study and screened for the presence of AIV antibodies by ELISA with the Influenza A Virus Antibody Test Kit (IDEXX, Hoofddorp, The Netherlands) targeting the nucleoprotein (NP) protein. The ELISA was performed according to instructions from the supplier. Antibody-positive samples were tested by hemagglutination inhibition (HI) test against a panel of AIV reference antigens provided by the European Union Reference Laboratory for Avian Influenza and Newcastle Disease at the time (Animal and Plant Health Agency) using 4 HA units of antigen. The antigens represented contemporary LPAI H5, H7, and clade 2.3.4.4b HPAI H5 viruses.

### 2.3. Sequencing and Consensus Sequence Generation

For whole genome sequencing, next generation sequencing was performed on eight-segment mixtures amplified in a single tube for each sample using the primers MBTuni12R and MBTuni13 [28], essentially as previously described [29]. Briefly, eight-segment amplification mixes were purified with High Pure PCR Product Purification Kit (Roche, Hvidovre, Denmark) and sequenced using the Nextera library preparation method and Illumina MiSeq paired-end 150 base pair sequencing. Fastq sequence data files were initially processed in CLC Genomics Workbench (version 8.0.2) (QIAGEN, Aarhus, Denmark). Reads were paired, trimmed, and subsequently aligned into contigs using the “de novo assembly” tool with default settings. Consensus sequences were extracted for each of the eight gene segments and used to identify the most similar reference sequence by BLASTN analyses in GenBank (NCBI, http://blast.ncbi.nlm.nih.gov//Blast.cgi) with default settings. These eight reference sequences were then used for mapping the sequencing reads, and consensus sequences were extracted. Additional HA and neuraminidase (NA) sequences from samples that were not full genome sequenced were generated by Sanger sequencing on PCR amplicons. Primer sequences are available upon request from the authors. The sequences generated in this study were submitted to GenBank with accession numbers denoted in Supplementary Table S5. Pairwise comparisons of nucleotide sequences and deduced amino acid sequences were performed using CLC Genomics Workbench (version 8.0.2) (QIAGEN, Aarhus, Denmark).

Concatenated sequences representing the gene segments polymerase basic protein 1 (PB1), polymerase acidic protein (PA), HA, NP, matrix protein (MP), and non-structural protein (NS) were constructed by the assembly of coding regions in the order from segment 1 to segment 8 using an in-house python script. Concatenated sequences representing all gene segments were constructed using the same in-house python script in the order from segment 1 to segment 8.

### 2.4. Data Selection

For phylogenetic and molecular dating analysis, related full-genome sequences were selected from the GenBank (NCBI, http://ncbi.nlm.nih.gov, last accessed 18 June 2020) and EpiFlu (GISAID, http://www.gisaid.org, last accessed 15 July 2020) databases. These included strains from the 2016–2017 H5N8 European outbreak, European H5N6 viruses from 2017–2018, and Asian H5N6 viruses from 2017–2018. European LPAI strains were also included based on percentage identity by nucleotide BLAST searches.

### 2.5. Bayesian Phylogenetic Analysis

For each AIV genome segment, phylogenetic trees were reconstructed using MrBayes (version 3.2.7a) with settings nst = mixed rates = invgamma [30]. MCMC was run for 100,000,000 cycles. Trees were visualized using FigTree (version 1.4.4) [31]. Tracer (version 1.7.1) [32] was used to check for convergence.

### 2.6. Molecular Clock Analysis

Molecular dating analysis was performed on the individual genome segments and on the concatenated H5N6 sequences. Model testing for each gene segment were performed with CLC Genomics Workbench (version 8.0.2) (QIAGEN, Aarhus, Denmark), which was also used to construct neighbor-joining trees with 1000 bootstrap replicates. The software TempEST (version 1.5.3) was used to check for the presence of temporal signal [33]. Molecular clock-based trees were reconstructed using BEAST2 (version 2.5.2) [34]. The concatenated sequences were analyzed using separate substitution models for each partition. All analyses used gamma distributed rates over sites, a strict molecular clock model, and the birth-death skyline serial model. The prior for the reproduction number was log normal, with a lower and upper value of 0 and 10, respectively, and with M = 0.0, S = 1.0 (meaning we are fairly certain the value is between 0 and 10), and 5 dimensions. The prior for “becomeUninfectiousRate” was log normal with M = 52.0 and Y=1.0, equalling to an average of 52 per year, i.e., an average time of being infectious is close to 1 week. The prior for the sampling proportion was set to log normal with M = 0.001 and S = 1.25. The prior for the clock rate was log normal with M = 0.001, S = 1.25 for individual gene segments, M = 0.006, S = 1.25 for concatenated data with six gene segments and M = 0.008, S = 1.25 for concatenated data with eight gene segments. Lastly, the prior for the origin was set to gamma with alpha = 0.5 and beta = 2.0. Other priors were left at default values. MCMC was run for 100,000,000 iterations and 10% pre-burn-in. Maximum clade credibility trees were subsequently generated with TreeAnnotator (version 2.6.2), with burn-in set to 50% and a posterior probability limit of 0.3 [34]. Trees were visualized using FigTree (version 1.4.4) [31]. Molecular clock analyses were run twice. Tracer (version 1.7.1) [32] was used to check the convergence.

## 3. Results

### 3.1. AIV Wild Bird Active Surveillance

In total, 1758 apparently healthy wild birds were included in the AIV active surveillance program in 2018–2019. Sampling was mainly performed from September to December, and the majority of birds were waterfowl, primarily mallard ducks. The samples were tested for the presence of AIV by real-time RT-PCR in a total of 489 pools. AIV was detected in 125 pools, of which LPAI H5 virus was detected in one pool from mallard ducks in 2018, and in four pools in 2019 of samples from common teals, Eurasian wigeons, northern shovelers, and mallard ducks, respectively. HPAI viruses were not detected in any of these birds. For further details, see Supplementary Figures S1 and S2 and Supplementary Tables S1 and S2.

### 3.2. AIV Wild Bird Passive Surveillance

A total of 259 wild birds found dead in the environment during 2018–2019 were tested for AIV. The sampling was distributed over the entire sampling period and all parts of Denmark (Supplementary Figures S3 and S4, Supplementary Tables S3 and S4). HPAI H5N6 virus was detected in 43 wild birds found in the period from 9 February 2018 to 4 January 2019 at 34 separate sampling occasions. In addition, AIV that was not of the H5 or H7 subtypes was detected in six birds on three sampling occasions.

**Figure 1:**
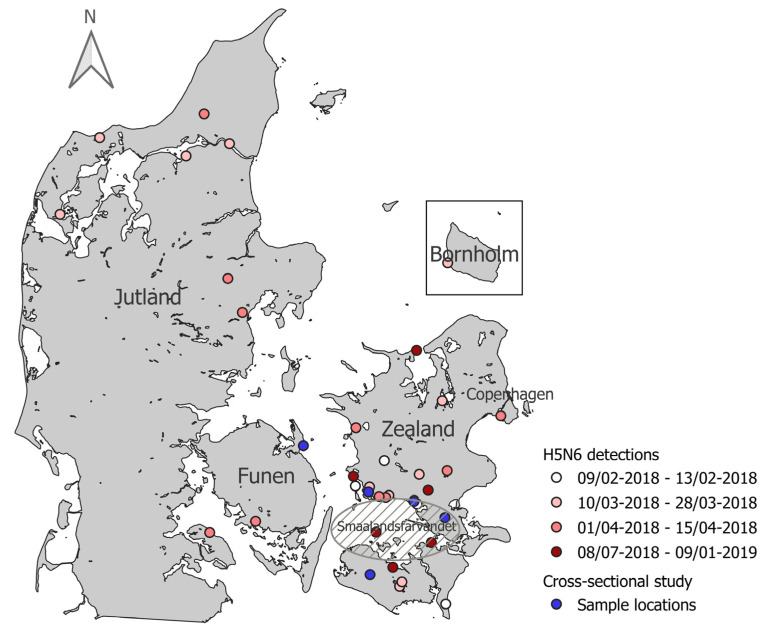
Geographic distribution of 2.3.4.4b H5N6 HPAIV detections in Denmark and sample locations of the cross-sectional study. Every white, pink, or red point represents one wild bird found dead and infected with H5N6. The gradient from white to red points represents when in the year the detections occurred. The blue points represent the locations where pheasants were collected for the cross-sectional study. Map made with QGIS [35]. Outline of Denmark from Kortforsyningen (www.kortforsyningen.dk, downloaded 26 April 2012).

The first detections of HPAI H5N6 virus in Denmark were in three white-tailed eagles found at different locations in the Southern Zealand area in February 2018, followed by detections in two white-tailed eagles in the northwestern part of Jutland in March 2018. Detections throughout March and April followed, mainly in common buzzards, and were scattered over most of the country with a high concentration of detections in the southeastern area of Denmark surrounding the “Smaalandsfarvandet”. There were no detections of HPAI virus positive birds in May and June 2018. The start of “late” sporadic detections was a single detection in a common eider found in the Southern part of “Smaalandsfarvandet” in early July. HPAI H5N6 virus was subsequently detected in a mute swan in early August and then further in mute swans, mallards, and pheasants in late August 2018. At the beginning of September 2018, infected pheasants, common eider, greylag goose, common eider, and mute swans were found. The last two detections were in a white-tailed eagle in December 2018 and a common buzzard on 4 January 2019. All the “late” detections were in birds found in the area surrounding “Smaalandsfarvandet”, except for the last positive bird that was found in Northern Zealand, approximately 100 km north of “Smaalandsfarvandet” (Figure 1).

### 3.3. HPAI Virus Prevalence in Clinically Healthy Wild Pheasants

Wild pheasants were sampled at five locations, Skælskør (*n* = 15), Præstø (*n* = 15), Næstved (*n* = 11), Søllested (*n* = 15), and Kerteminde (*n* = 13) during January to March 2019 (Figure 1). Antibodies against AIV were detected in the serum from one wild pheasant from Skælskør by ELISA and subtyped as H5 by HI-test against H5N8 and H5N5 clade 2.3.4.4 antigens (titers 64). No reaction against other antigens in the HI-test was detected. Antibodies were not detected in any of the other blood samples, and AIV was not detected by PCR in any of the swab samples.

### 3.4. Phylogenetic Analyses

Whole genome sequences were obtained by NGS from 16 HPAI H5N6-positive wild birds detected during the passive surveillance for AIV in Denmark. Full length HA (or both HA and NA) sequences were obtained by Sanger sequencing from four additional HPAI H5N6-positive wild birds (Table 1). Bayesian phylogenetic analyses (Supplementary Figure S5) for each gene segment revealed the Danish HPAI H5N6 viruses to be closely related to each other with nucleotide sequence identities ranging from 98.94–100% and amino acid sequence identities ranging from 97.88–100%. The topology of the trees of each of the eight segments (Supplementary Figure S5) were highly similar and thus indicated that reassortment had not occurred within the Danish H5N6 viruses.

**Figure 2:**
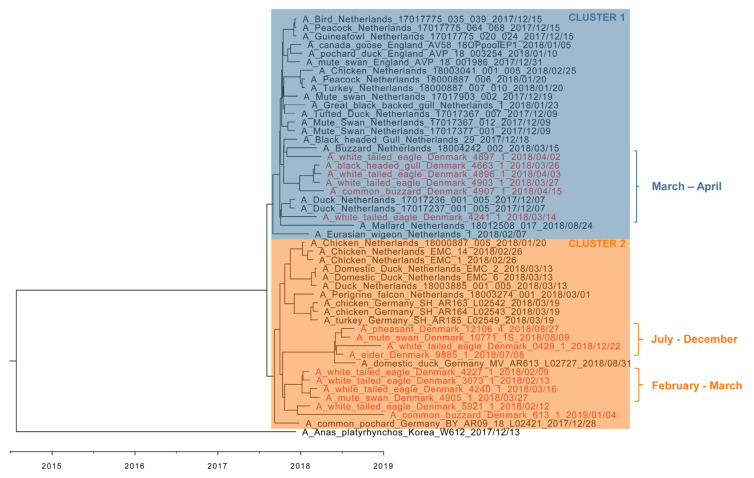
BEAST analysis on concatenated dataset including all European clade 2.3.4.4b H5N6 HPAIV detected in 2017–2019. All gene segments of the influenza viruses included in the analysis were concatenated. The H5N6 viruses isolated in Denmark are in red with the rest being in black. The viruses within the clock tree can be divided into two major clusters, which have been denoted as cluster 1 (blue) and cluster 2 (orange). The scale bar depicts a timeline.

Since the eight genome segments from the Danish HPAI H5N6 viruses share their evolutionary history, we concatenated all gene segment sequences for further analysis. Phylogenetic analysis of the 16 Danish full-genome sequenced viruses along with contemporary European HPAI H5N6 virus sequences and representative reference viruses showed that the sequences fall in two clusters (Figure 2). The Danish sequences within “cluster 1” were obtained from six wild birds found in March and April 2018. Four of these were found in Southwest Zealand, one in Southern Jutland and one in Northwestern Jutland. “Cluster 2” contained sequences from ten wild birds collected in the Southern part of Zealand and nearby islands, together with the last detected HPAI H5N6-positive bird from Northern Zealand. Within cluster 2, a subcluster was identified including sequences obtained from birds collected in February and March in the “Smaalandsfarvandet” area. Viral sequences from birds collected in July to December in the same area formed another subcluster that was more closely related to the Danish sequences originating from the common buzzard from January 2019 and a white-tailed eagle from February 2018 and to German HPAI H5N6 sequences. Viruses detected in birds from the “Smaalandsfarvandet” area were thus dispersed in both clusters, but all “late” HPAI H5N6 virus findings belonged to cluster 2. Notably, a German H5N6 virus detected in August (A/domestic duck/Germany-MV/AR163/L02727/2018) was in cluster 2 along with the late-2018 Danish viruses, while the H5N6 virus found in the Netherlands (A/Mallard/Netherlands/18012508-017/2018) in the same period was located in cluster 1.

It has previously been reported that the European HPAI H5N6 viruses from late 2017–2018 were related to HPAI H5N8 clade 2.3.4.4 viruses from 2016–2017, but containing PB2 and neuraminidase (NA) segments from LPAI viruses [17,35]. To make a comparison of clade 2.3.4.4b HPAI viruses detected in Europe, we analyzed concatenated sequences excluding the PB2 and NA gene segments representing the European H5N6 and H5N8 full-genome sequenced viruses detected in birds during 2017–2019 and 2016–2017, respectively (Supplementary Figure S6). The topology of the tree shows a relatedness of the Danish and European HPAI H5N6 viruses to European HPAI H5N8 viruses from 2016–2017 in all included gene segments, but with the H5N6 viruses grouping into a separate cluster.

### 3.5. Molecular Dating

To shed light on evolutionary events, molecular dating analysis was performed. Estimation of time to the most recent common ancestor (tMRCA) shows that the common ancestor of the included European H5N6 HA gene segments was present around July 2017 (Supplementary Figure S7, HA) (Table 1, HA, node 1).

The Danish NA segments from HPAI H5N6 viruses cluster with European H5N6 viruses from the same period, similar to the H5 segment (Supplementary Figure S7, NA). Thus, similar to these, they are closely related to the European LPAI N6 viruses. The clock-based evolutionary analysis estimates a common ancestor circulating in January 2017 (Table 1, NA, node 1). The closest relation to the 2017–2019 European N6 as well as the Greek and Asian N6 segments is the LPAI H4N6 A/mallard duck/Georgia/3/2016.

We also performed molecular dating analysis to elucidate the emergence of the PB2 segment (Supplementary Figure S7, PB2). The tMRCA for the late 2017–2019 Danish and other European H5N6 PB2 segments are estimated to have circulated in April 2017 (Table 1, PB2, node 1). The phylogenetic analyses of the PB2 segments reveal a close relation to European LPAI circulating in 2011–2012 with the tMRCA around February 2010 (Table 1, PB2, node 3). The closest related isolate is estimated to be a H5N2 virus found in the Netherlands in 2011 (A/mallard duck/Netherlands/32/2011).

### 3.6. Zoonotic Potential

To investigate the zoonotic potential of the Danish HPAI H5N6 viruses, the viral genome sequences were analyzed for a range of markers (Supplementary Table S6). Mutations characteristic for clade 2.3.4.4b H5NX viruses were identified, but none of these indicate increased zoonotic potential for the Danish H5N6 viruses; however, amino acid residues encoding desensitization to oseltamivir were observed.

## 4. Discussion

At the end of 2017, several European countries were affected by HPAI H5N6 outbreaks in wild birds and poultry. The vast majority of detections were registered in the period from February to April 2018. In April, detections were reported from Finland, Sweden, and Denmark in wild birds. The following detection was reported from the United Kingdom in a wild bird in mid-June. While HPAI H5N6 virus was also reported in August in Germany in captive birds [36] and the Netherlands in wild birds [37], no further detections of H5N6 viruses in 2018 were reported after August in any other European countries, besides Denmark [16]. In Denmark, there were no detections in poultry or in clinically healthy wild birds in 2018–2019. However, HPAI H5N6 viruses were detected in 43 dead wild birds in the AIV passive surveillance program. Detections were mainly in the period from February to April, but unique to the Danish epizootic were sporadic “late” detections that continued until 4 January 2019. These findings raised the question of whether the late detections were caused by continuous circulation of the H5N6 virus locally or by reintroduction to Denmark.

To examine the genetic evolution of HPAI H5N6 viruses detected in Denmark, full genome sequences of 16 Danish HPAI H5N6 viruses were obtained. Our phylogenetic analysis of the Danish H5N6 viruses revealed these to be genetically similar to each other as well as other European H5N6 clade 2.3.4.4b viruses detected in 2017–2018. The virus likely emerged from European HPAI H5N8 viruses from 2016–2017 containing the PA II gene segment variant observed in Dutch viruses [13]. The virus subsequently obtained the N6 and PB2 gene segments from Eurasian LPAI viruses by reassortment. These observations are in concurrence with previously published results on European H5N6 viruses [4,17,36]. The novel HPAI H5NX clade 2.3.4.4 viruses have previously been reported to originate from reassortment of influenza viruses at breeding and molting grounds in Siberia, which then disseminate to other geographic regions, including Europe [3]. Our analyses are in agreement with the previous hypothesis that H5N6 viruses in Europe sampled between 2017–2019 may have evolved by a reassortment event in 2017 between a European HPAI H5N8 virus and LPAI HXN6 viruses at Siberian breeding sites [4,17]. The newly reassorted virus subsequently spread by long-distance migratory birds to their European wintering areas.

To determine the evolutionary history of the Danish H5N6 viruses, we performed molecular dating analysis and calculated the tMRCA. It was estimated that the common ancestor for the HA and NA gene segments of European HPAI H5N6 viruses circulated in July 2017 and January 2017, respectively. These estimates are in agreement with Beerens et al. estimating tMRCA to be September 2017 for HA and April 2017 for NA [4]. Notably, the concatenated molecular clock tree shows a clear separation of European H5N6 viruses into two different clusters, suggesting at least two introductions of phylogenetically distinct virus into Denmark, one occurring around February 2018 and another around March 2018. The second wave of H5N6 detections in the latter half of 2018 were potentially a result of the continuous presence and circulation in a reservoir of the earlier introduction of H5N6 from February 2018, as the viruses were grouped in the same cluster. An alternative hypothesis is that the second wave was caused by a reintroduction of H5N6 into Danish grounds. While H5N6 detections were recorded in August in Denmark, Germany, and the Netherlands, only the Danish and German cases cluster together. This indicates that at least two slightly different H5N6 variants were circulating in the latter half of 2018, one possibly having persisted in or close to the Netherlands and another in or close to Denmark and the northern part of Germany. Beerens et al. suggest that the Dutch H5N6 virus detected in August persisted in their local wild bird populations from an earlier introduction [37].

The late 2018 and early 2019 Danish cases of H5N6-infected dead wild birds were all found in the same region, “Smaalandsfarvandet”. Five of the H5N6 virus detections from August and September 2018 were in wild pheasants found dead. In Denmark, the production of pheasants is mainly for hunting purposes. They are released into the wild between 1 April and 31 August, and otherwise roam freely until they are shot or captured for the breeding of next year’s game pheasants. During this period, the pheasants will probably remain close to the site of release, as they are often still supplied with feed. Thus, they may seek contact with free-range poultry in the area and by that, contribute to outbreaks in poultry. We hypothesize that HPAI virus spillover infections to residential birds, in this case pheasants, could have played a role in the continuous circulation of H5N6 viruses. From January to March 2019, wild pheasants from five different locations in or around the area of “Smaalandsfarvandet” were therefore collected to test this hypothesis. While virus was not detected in the pheasants, antibodies were detected in one pheasant, indicating that the bird had been infected with a clade 2.3.4.4 H5 influenza virus. It has previously been reported that certain LPAI strains result in persistent subclinical infection in pheasants and are shed for a prolonged period of time resulting in the occurrence of antigenic drift variants [19,20]. The transmission of HPAI viruses to game-bred pheasants could therefore play a role in the persistence of AIV in a region. Our results support the previous indications that pheasants may act as a reservoir of AIV viruses, but further studies are needed. The pathogenesis and molecular adaption of HPAI in pheasants is currently being investigated in ongoing experimental studies. Other resident wild bird species may also have contributed to the maintenance of the HPAI H5N6 viruses. Active surveillance for AIV in 1758 clinically healthy wild birds was conducted in Denmark during the H5N6 late epizootic. Although HPAI viruses were not detected in any of these birds, the persistence of HPAI H5N6 viruses in a low proportion of healthy wild birds cannot be excluded. Interestingly, significantly fewer LPAI H5 viruses were detected in wild birds in Denmark in 2018 and 2019. This is in contrast to earlier years of active surveillance in Denmark, where much higher rates of LPAI H5 viruses were detected, e.g., in 2017, 21 LPAI H5 viruses were detected [38,39]. We speculate that the HPAI H5H8 and H5N6 epizootics in 2017–2019 may have imposed increased population immunity toward the H5 subtype in the wild bird population. An extensive serosurveillance program is needed to confirm this assumption.

Several laboratory-confirmed cases of human infections with H5N6 virus, including seven deaths, have been reported from China since 2014 (World Health Organization, 2019). The European 2017–2019 HPAI H5N6 viruses are only distantly related to the Asian H5N6 viruses that have caused human infections, but they seem to have evolved from the H5N8 2016–2017 viruses by reassortment with wild host reservoir LPAI viruses and, furthermore, not to possess the characteristic markers for human infection potential. Even so, constant surveillance is necessary to characterize the ongoing evolution of the virus in wild birds to ensure that this pathogen does not become a substantial burden for domestic poultry production, or a threat to public health.

## Figures and Tables

**Table 1:** Estimated tMRCA on HA, NA, and PB2 and 95% HPD intervals have been calculated using BEAST analysis. The posterior probabilities are of the branch before the node. Node numbers are depicted in Supplementary Figure S7.

	Node	tMRCA	95% HPD Interval	Posterior Probability	Origin
PB2	1	April 2017	January 2017–August 2017	1.0000	European LPAI
	2	May 2010	November 2009–November 2010	0.5341	
	3	February 2010	August 2009–August 2010	0.9228	
HA	1	July 2017	May 2017–September 2017	0.5786	H5N8 HPAI 2016–2017
	2	June 2017	March 2017–August 2017	1.0000	
	3	September 2016	July 2016–November 2016	0.311	
NA	1	January 2017	October 2016–May 2017	0.7741	H5N6 HPAI 2017
	2	December 2016	August 2016–April 2017	1.0000	European LPAI
	3	July 2015	December 2014–January 2016	1.0000	

## Data Availability

Newly determined sequences were deposited in Genbank (Accession no. can be found in Supplementary Table S5).

## Supplementary Materials

The following are available online at https://www.mdpi.com/article/10.3390/v13061052/s1, Figure S1: Active surveillance for avian influenza virus in clinically healthy wild birds, Denmark, 2018. Figure S2: Active surveillance for avian influenza virus in clinically healthy wild birds, Denmark, 2019. Figure S3: Sampling locations for avian influenza virus wild bird passive surveillance, Denmark, 2018. Figure S4: Sampling locations for avian influenza virus wild bird passive surveillance, Denmark, 2019. Figure S5: Bayesian phylogenetic analysis performed on each gene segments of H5N6 viruses detected in Denmark 2018–2019. Figure S6: BEAST analysis on concatenated dataset including European clade 2.3.4.4b H5N6 viruses detected in 2017–2019 and representative European clade 2.3.4.4b H5N8 viruses detected in 2016–2017. Figure S7: BEAST analysis on HA, NA, and PB2 gene segments. Numbers above the nodes represent tMRCA. Table S1: Results from active surveillance for avian influenza virus in clinically healthy wild birds, Denmark 2018. Table S2: Results from active surveillance for avian influenza virus in clinically healthy wild birds, Denmark, 2019. Table S3: Results of avian influenza virus wild bird passive surveillance, Denmark, 2018. Table S4: Results of avian influenza virus wild bird passive surveillance, Denmark, 2019. Table S5: Details of the Danish clade 2.3.4.4b H5N6 HPAIV that have been sequenced in this study. Table S6: Risk signatures. Table S7: Acknowledgement of laboratories providing sequences to the GISAID EpiFlu database and NCBI Genbank database.
