# Supplementary material for: Molecular Characterization of Highly Pathogenic Avian Influenza Viruses H5N6 Detected in Denmark in 2018–2019
Source: Viruses. 2021 Jun 2;13(6):1052. doi: 10.3390/v13061052 (PMC8226499; doi:10.3390/v13061052)
Supplement: Supplementary file 1 [file viruses-13-01052-s001.zip › viruses-1224460-supplementary.pdf]

viruses

MDPI

Molecular Characterization of Highly Pathogenic Avian Influenza Viruses H5N6 Detected in Denmark 2018-2019

Supplementary Material

Figure S1. Active surveillance for avian influenza virus in clinically healthy wild bird, Denmark, 2018. Birds were tested for avian influenza virus in pools of cloacal swab samples from up to five birds of the same species, sampled at the same time and location. Sampling locations are shown for each pool, with points distributed in concentric circles with origin in the center of the circles. Map made with QGIS (version 3.14.1) (www.qgis.org). Based on map of Denmark from Kortforsyningen (www.kortforsyningen.dk downloaded 26 April 2012).

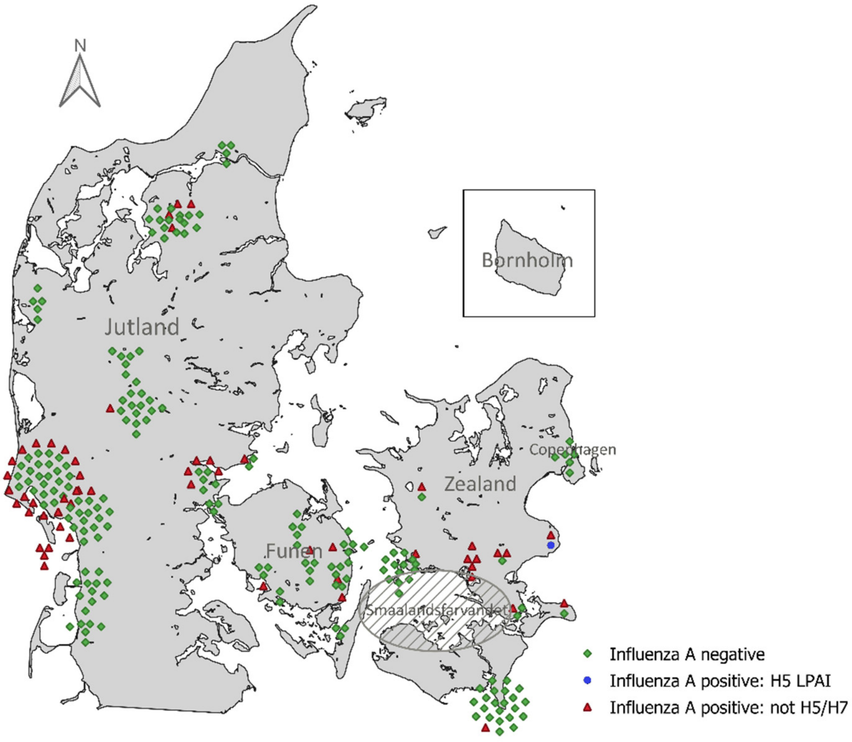

Liang, Y.; Krog, J.S.; Ryt-Hansen, P.; Pedersen, A.G.; Kvisgaard, L.K.; Holm, E.; Nielsen, P.D.; Hammer, A.S.; Madsen, J.J.; Thorup, K.; Larsen, L.E.; Hjulsager, C.K. Molecular Characterization of Highly Pathogenic Avian Influenza Viruses H5N6 Detected in Denmark in 2018–2019. *Viruses* **2021**

Figure S2. Active surveillance for avian influenza virus in clinically healthy wild bird, Denmark, 2019. Birds were tested for avian influenza virus in pools of cloacal swab samples from up to five birds of the same species, sampled at the same time and location. Sampling locations are shown for each pool, with points distributed in concentric circles with origin in the center of the circles. Map made with QGIS (version 3.14.1) (www.qgis.org). Based on map of Denmark from Kortforsyningen (www.kortforsyningen.dk downloaded 26 April 2012).

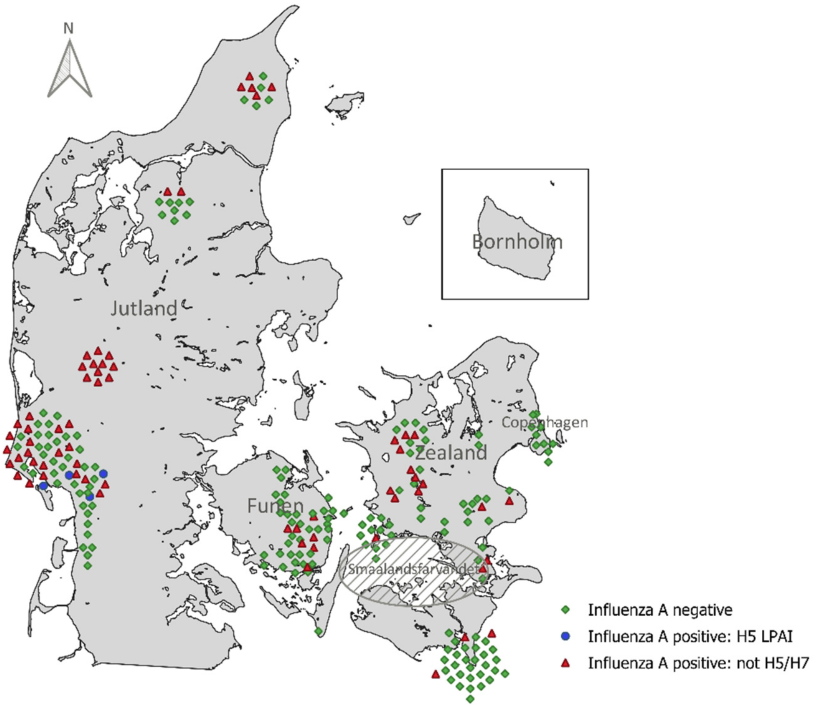

Figure S3: Sampling locations for avian influenza virus wild bird passive surveillance, Denmark, 2018. Map made with QGIS (version 3.14.1) (www.qgis.org). Based on map of Denmark from Kortforsyningen (www.kortforsyningen.dk downloaded 26 April 2012).

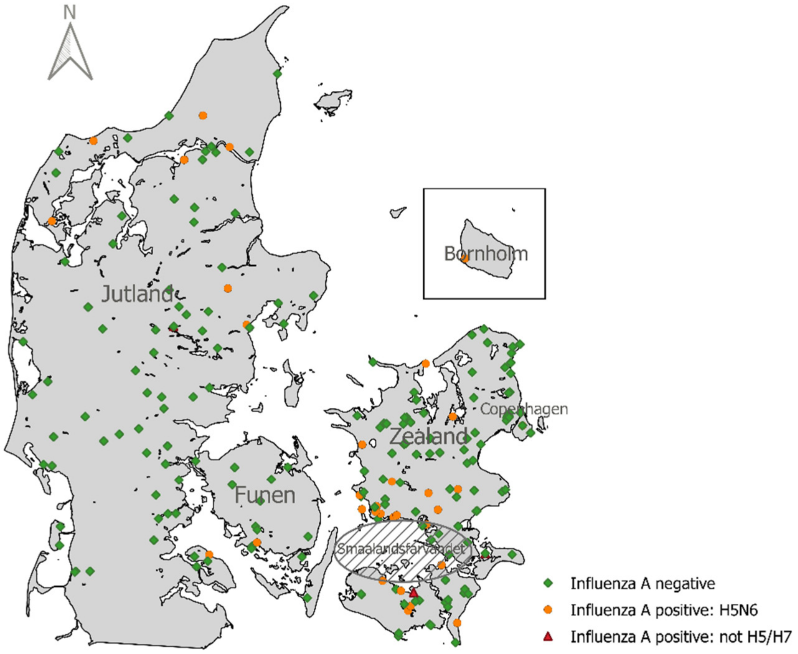

Figure S4: Sampling locations for avian influenza virus wild bird passive surveillance, Denmark, 2019. Map made with QGIS (version 3.14.1) (www.qgis.org). Based on map of Denmark from Kortforsyningen (www.kortforsyningen.dk downloaded 26 April 2012).

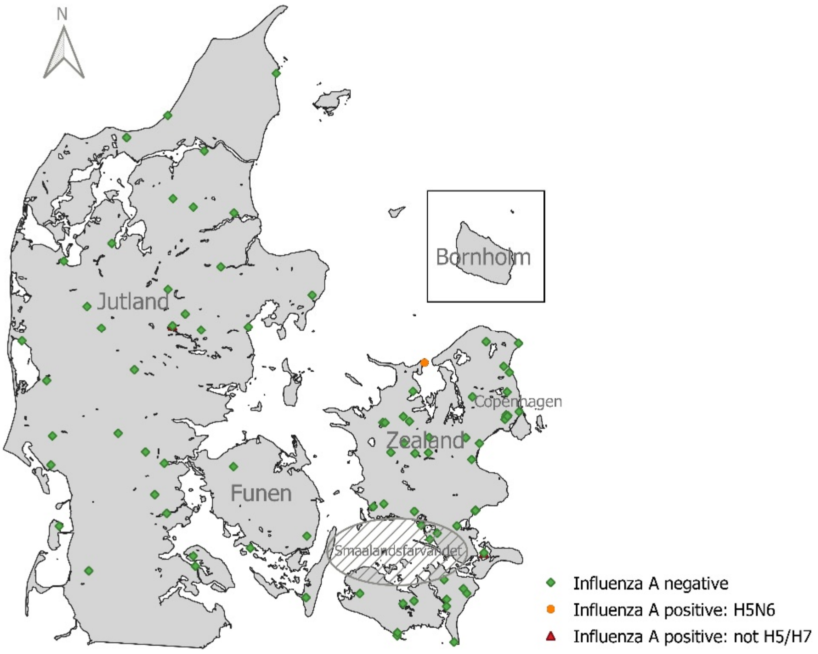

Figure S5. Bayesian phylogenetic analysis performed on each gene segments of H5N6 viruses detected in Denmark 2018-2019. Branch labels represent posterior probabilities. Scale bars indicate nucleotide substitutions per site.

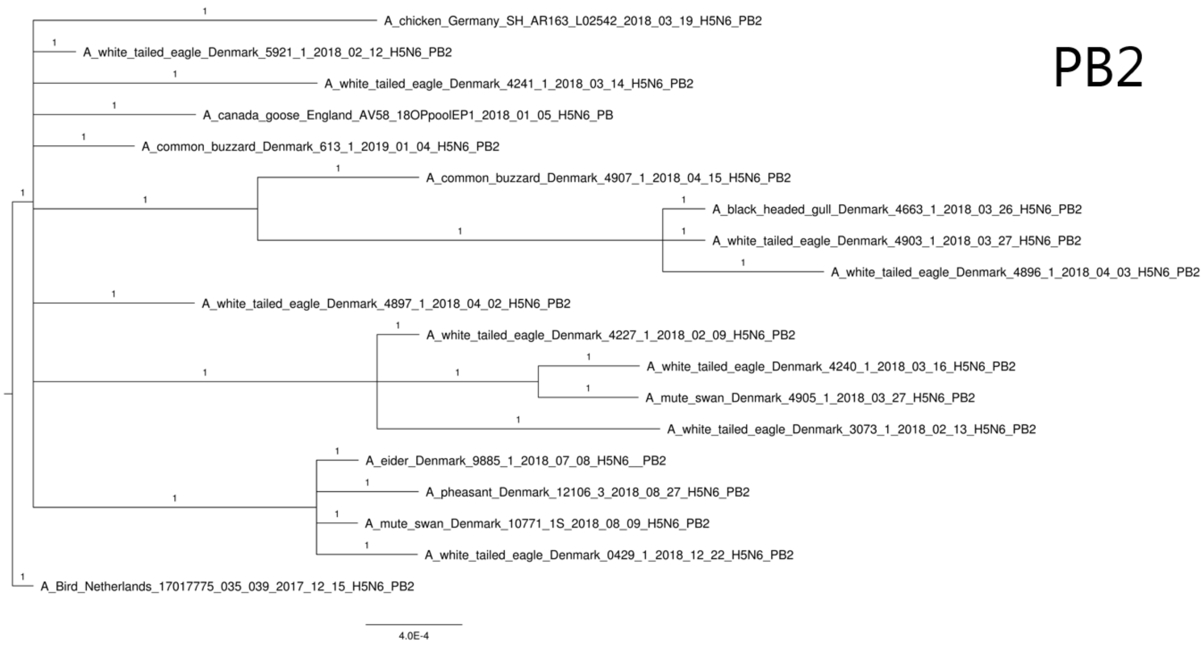

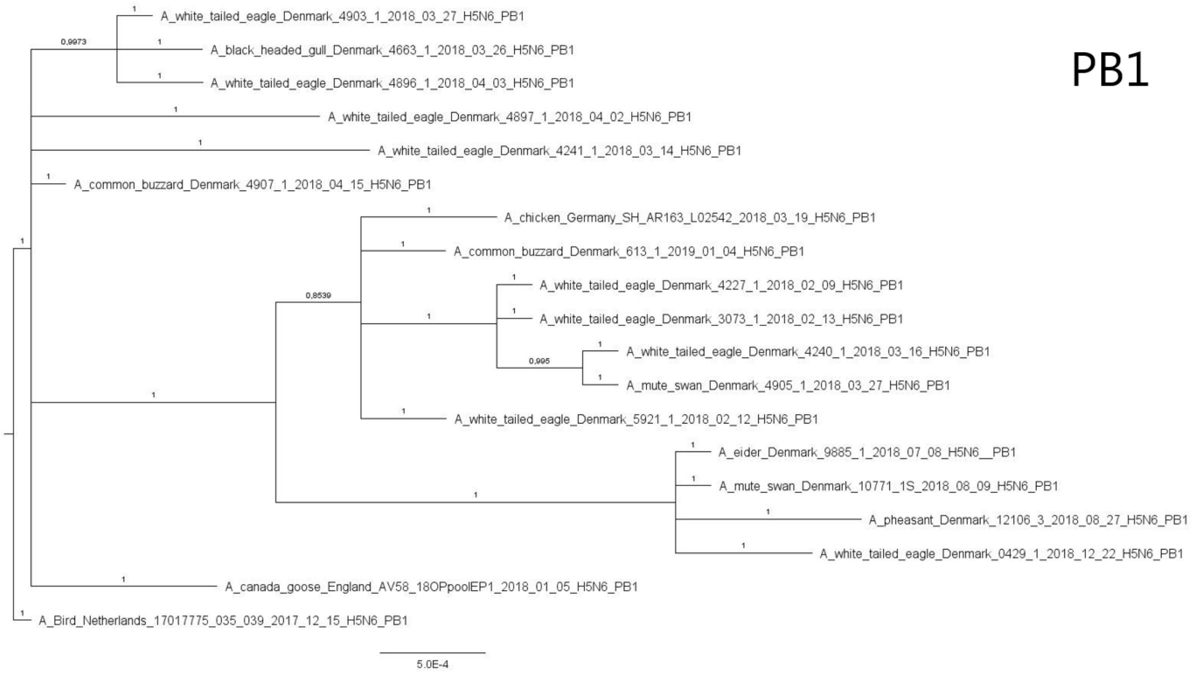

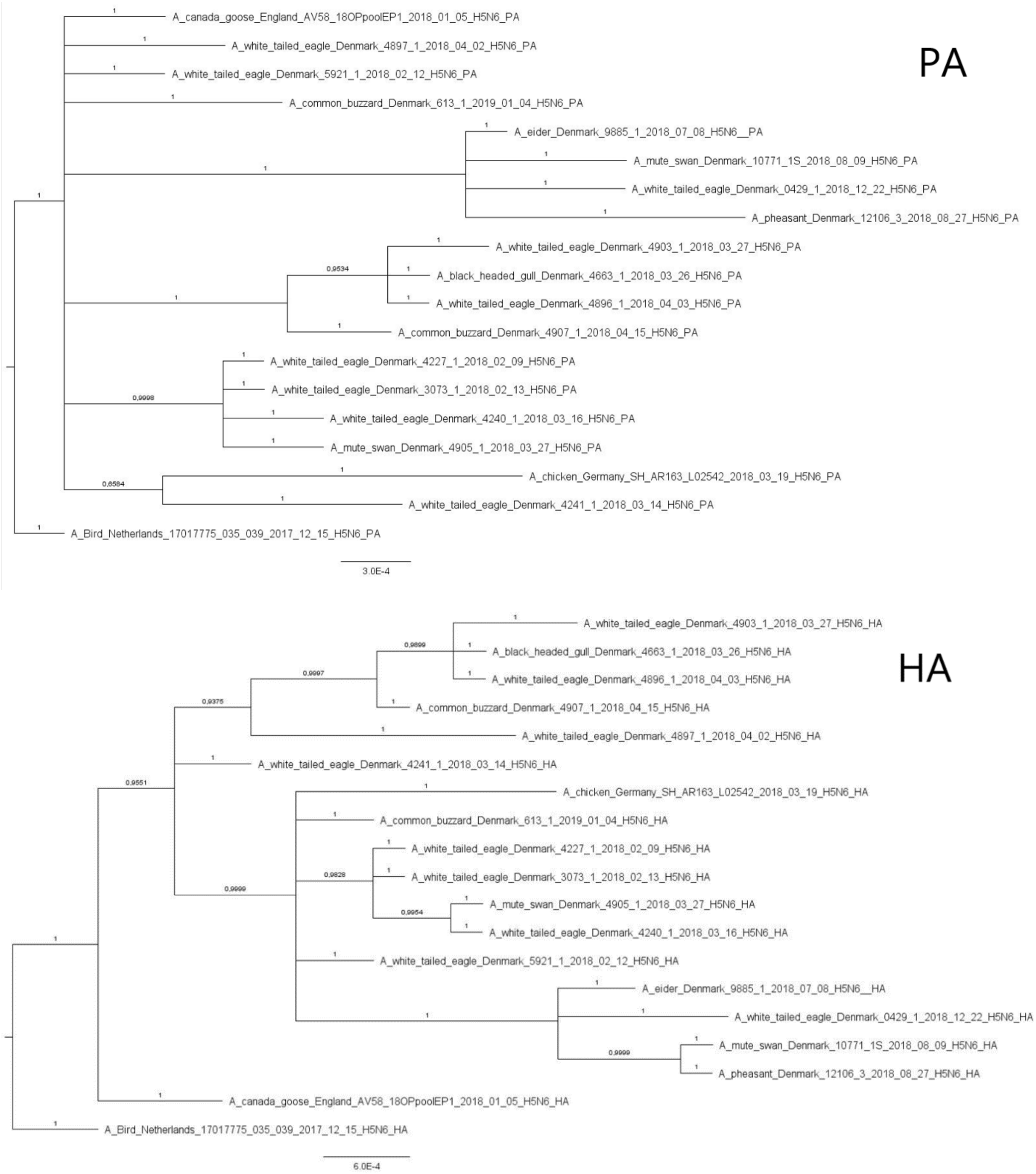

Liang, Y.; Krog, J.S.; Ryt-Hansen, P.; Pedersen, A.G.; Kvisgaard, L.K.; Holm, E.; Nielsen, P.D.; Hammer, A.S.; Madsen, J.J.; Thorup, K.; Larsen, L.E.; Hjulsager, C.K. Molecular Characterization of Highly Pathogenic Avian Influenza Viruses H5N6 Detected in Denmark in 2018–2019. *Viruses* **2021**

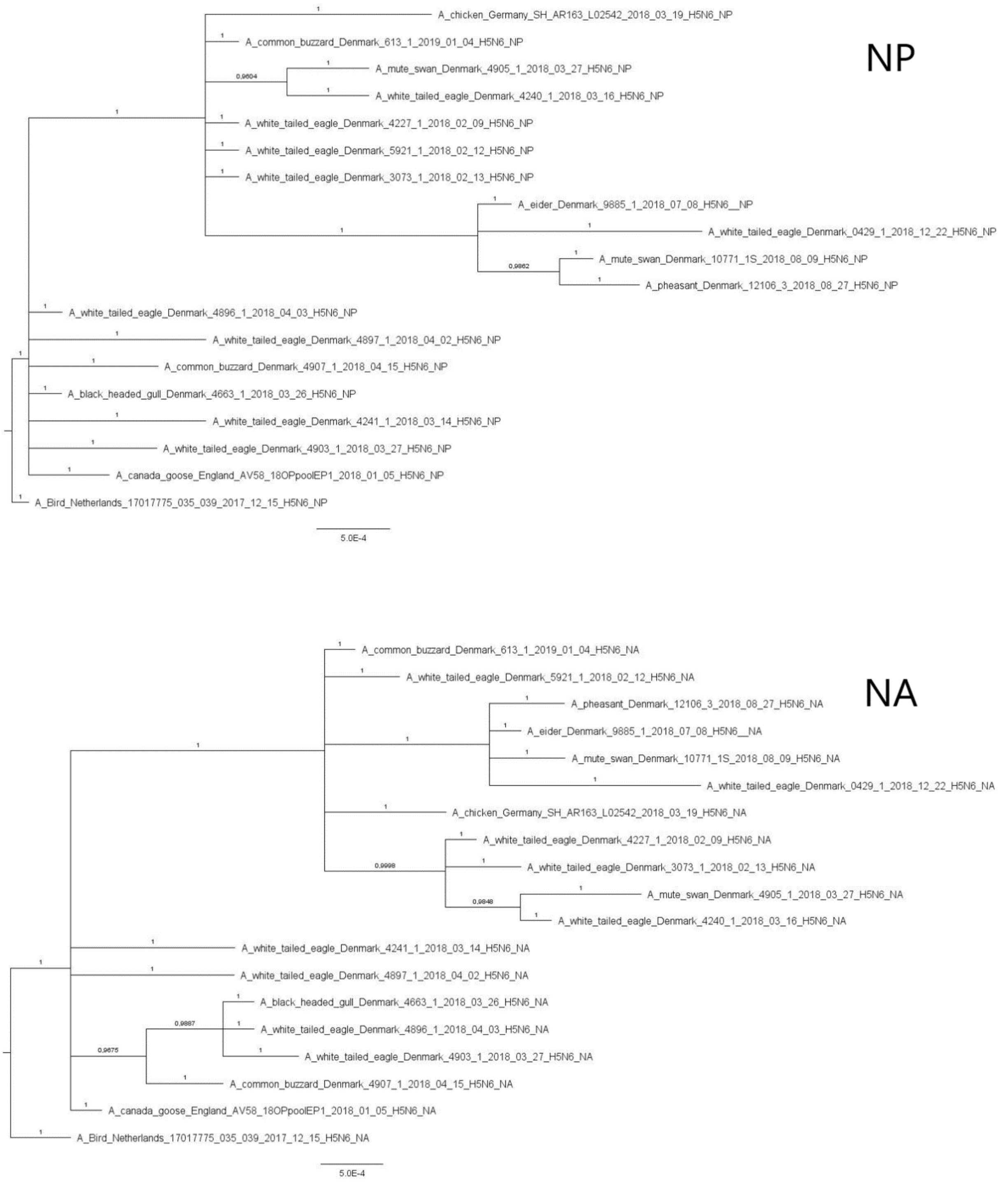
NP
A_chicken_Germany_SH_AR163_L02542_2018_03_19_H5N6_NP
A_common_buzzard_Denmark_613_1_2019_01_04_H5N6_NP
0.9604
A_mute_swan_Denmark_4905_1_2018_03_27_H5N6_NP
A_white_tailed_eagle_Denmark_4240_1_2018_03_16_H5N6_NP
A_white_tailed_eagle_Denmark_4227_1_2018_02_09_H5N6_NP
A_white_tailed_eagle_Denmark_5921_1_2018_02_12_H5N6_NP
A_white_tailed_eagle_Denmark_3073_1_2018_02_13_H5N6_NP
A_eider_Denmark_9885_1_2018_07_08_H5N6__NP
A_white_tailed_eagle_Denmark_0429_1_2018_12_22_H5N6_NP
0.9862
A_mute_swan_Denmark_10771_1S_2018_08_09_H5N6_NP
A_pheasant_Denmark_12106_3_2018_08_27_H5N6_NP
A_white_tailed_eagle_Denmark_4896_1_2018_04_03_H5N6_NP
A_white_tailed_eagle_Denmark_4897_1_2018_04_02_H5N6_NP
A_common_buzzard_Denmark_4907_1_2018_04_15_H5N6_NP
A_black_headed_gull_Denmark_4663_1_2018_03_26_H5N6_NP
A_white_tailed_eagle_Denmark_4241_1_2018_03_14_H5N6_NP
A_white_tailed_eagle_Denmark_4903_1_2018_03_27_H5N6_NP
A_canada_goose_England_AV58_18OPpoolEP1_2018_01_05_H5N6_NP
A_Bird_Netherlands_17017775_035_039_2017_12_15_H5N6_NP
5.0E-4
NA
A_common_buzzard_Denmark_613_1_2019_01_04_H5N6_NA
A_white_tailed_eagle_Denmark_5921_1_2018_02_12_H5N6_NA
A_pheasant_Denmark_12106_3_2018_08_27_H5N6_NA
A_eider_Denmark_9885_1_2018_07_08_H5N6__NA
A_mute_swan_Denmark_10771_1S_2018_08_09_H5N6_NA
A_white_tailed_eagle_Denmark_0429_1_2018_12_22_H5N6_NA
A_chicken_Germany_SH_AR163_L02542_2018_03_19_H5N6_NA
A_white_tailed_eagle_Denmark_4227_1_2018_02_09_H5N6_NA
0.9998
A_white_tailed_eagle_Denmark_3073_1_2018_02_13_H5N6_NA
A_mute_swan_Denmark_4905_1_2018_03_27_H5N6_NA
0.9848
A_white_tailed_eagle_Denmark_4240_1_2018_03_16_H5N6_NA
A_white_tailed_eagle_Denmark_4241_1_2018_03_14_H5N6_NA
A_white_tailed_eagle_Denmark_4897_1_2018_04_02_H5N6_NA
A_black_headed_gull_Denmark_4663_1_2018_03_26_H5N6_NA
0.9887
A_white_tailed_eagle_Denmark_4896_1_2018_04_03_H5N6_NA
0.9675
A_white_tailed_eagle_Denmark_4903_1_2018_03_27_H5N6_NA
A_common_buzzard_Denmark_4907_1_2018_04_15_H5N6_NA
A_canada_goose_England_AV58_18OPpoolEP1_2018_01_05_H5N6_NA
A_Bird_Netherlands_17017775_035_039_2017_12_15_H5N6_NA
5.0E-4

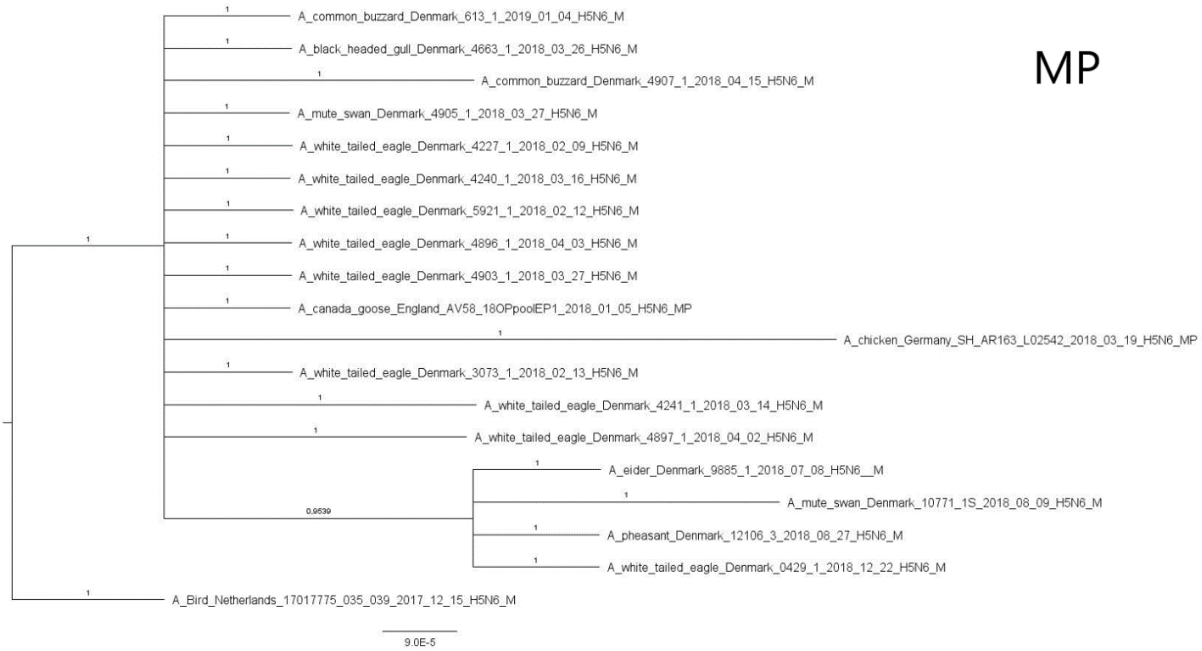
MP
A_common_buzzard_Denmark_613_1_2019_01_04_H5N6_M
A_black_headed_gull_Denmark_4663_1_2018_03_26_H5N6_M
A_common_buzzard_Denmark_4907_1_2018_04_15_H5N6_M
A_mute_swan_Denmark_4905_1_2018_03_27_H5N6_M
A_white_tailed_eagle_Denmark_4227_1_2018_02_09_H5N6_M
A_white_tailed_eagle_Denmark_4240_1_2018_03_16_H5N6_M
A_white_tailed_eagle_Denmark_5921_1_2018_02_12_H5N6_M
A_white_tailed_eagle_Denmark_4896_1_2018_04_03_H5N6_M
A_white_tailed_eagle_Denmark_4903_1_2018_03_27_H5N6_M
A_canada_goose_England_AV58_18OPpoolEP1_2018_01_05_H5N6_MP
A_chicken_Germany_SH_AR163_L02542_2018_03_19_H5N6_MP
A_white_tailed_eagle_Denmark_3073_1_2018_02_13_H5N6_M
A_white_tailed_eagle_Denmark_4241_1_2018_03_14_H5N6_M
A_white_tailed_eagle_Denmark_4897_1_2018_04_02_H5N6_M
A_eider_Denmark_9885_1_2018_07_08_H5N6__M
A_mute_swan_Denmark_10771_1S_2018_08_09_H5N6_M
A_pheasant_Denmark_12106_3_2018_08_27_H5N6_M
A_white_tailed_eagle_Denmark_0429_1_2018_12_22_H5N6_M
A_Bird_Netherlands_17017775_035_039_2017_12_15_H5N6_M
0.9539
9.0E-5

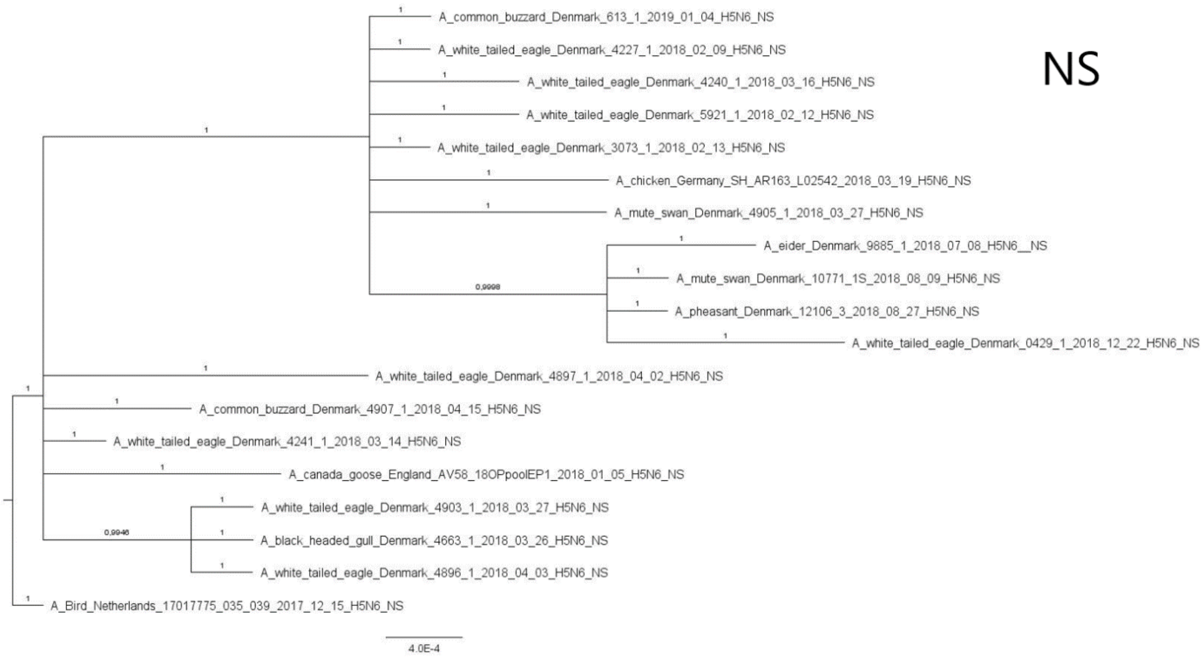
NS
A_common_buzzard_Denmark_613_1_2019_01_04_H5N6_NS
A_white_tailed_eagle_Denmark_4227_1_2018_02_09_H5N6_NS
A_white_tailed_eagle_Denmark_4240_1_2018_03_16_H5N6_NS
A_white_tailed_eagle_Denmark_5921_1_2018_02_12_H5N6_NS
A_white_tailed_eagle_Denmark_3073_1_2018_02_13_H5N6_NS
A_chicken_Germany_SH_AR163_L02542_2018_03_19_H5N6_NS
A_mute_swan_Denmark_4905_1_2018_03_27_H5N6_NS
A_eider_Denmark_9885_1_2018_07_08_H5N6__NS
A_mute_swan_Denmark_10771_1S_2018_08_09_H5N6_NS
A_pheasant_Denmark_12106_3_2018_08_27_H5N6_NS
A_white_tailed_eagle_Denmark_0429_1_2018_12_22_H5N6_NS
A_white_tailed_eagle_Denmark_4897_1_2018_04_02_H5N6_NS
A_common_buzzard_Denmark_4907_1_2018_04_15_H5N6_NS
A_white_tailed_eagle_Denmark_4241_1_2018_03_14_H5N6_NS
A_canada_goose_England_AV58_18OPpoolEP1_2018_01_05_H5N6_NS
A_white_tailed_eagle_Denmark_4903_1_2018_03_27_H5N6_NS
A_black_headed_gull_Denmark_4663_1_2018_03_26_H5N6_NS
A_white_tailed_eagle_Denmark_4896_1_2018_04_03_H5N6_NS
A_Bird_Netherlands_17017775_035_039_2017_12_15_H5N6_NS
0.9998
0.9946
4.0E-4

Figure S6. BEAST analysis on concatenated dataset including European clade 2.3.4.4b H5N6 viruses detected in 2017-2019 and representative European clade 2.3.4.4b H5N8 viruses detected in 2016-2017. The gene segments PB1, PA, HA, NP, MP and NS of the influenza viruses included in the analysis were concatenated in that order. The H5N6 viruses isolated in Denmark are in red, the representative H5N8 viruses are in green with the remaining H5N6 viruses in black. Posterior probabilities are denoted above the branches. The scale bar depicts a timeline.

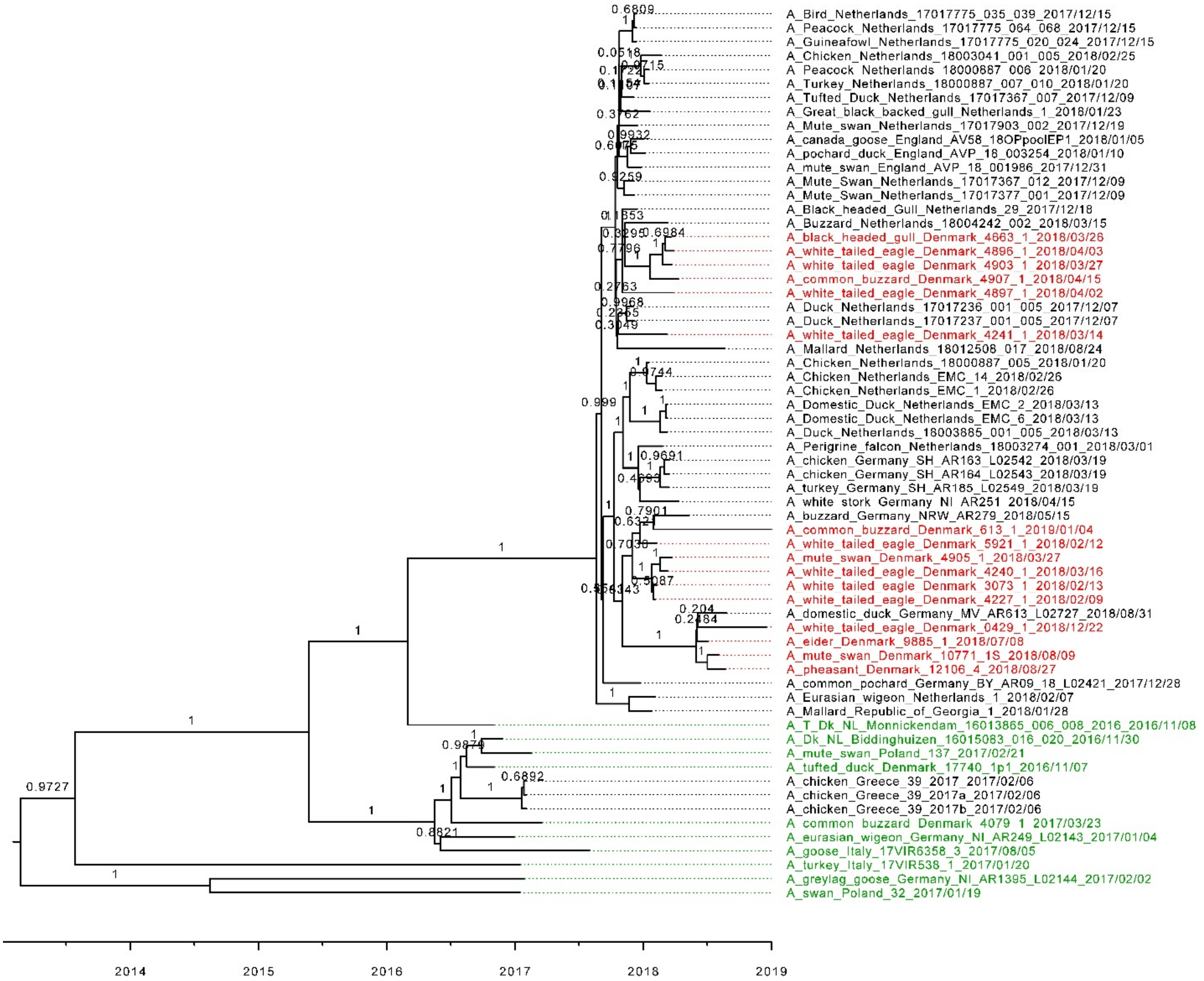

Liang, Y.; Krog, J.S.; Ryt-Hansen, P.; Pedersen, A.G.; Kvisgaard, L.K.; Holm, E.; Nielsen, P.D.; Hammer, A.S.; Madsen, J.J.; Thorup, K.; Larsen, L.E.; Hjulsager, C.K. Molecular Characterization of Highly Pathogenic Avian Influenza Viruses H5N6 Detected in Denmark in 2018–2019. *Viruses* **2021**

Figure S7. BEAST analysis on HA, NA and PB2 gene segments. Numbers above the nodes represent tMRCA. The HPAI H5N6 viruses from Denmark are in red. The scale bar depicts a timeline.

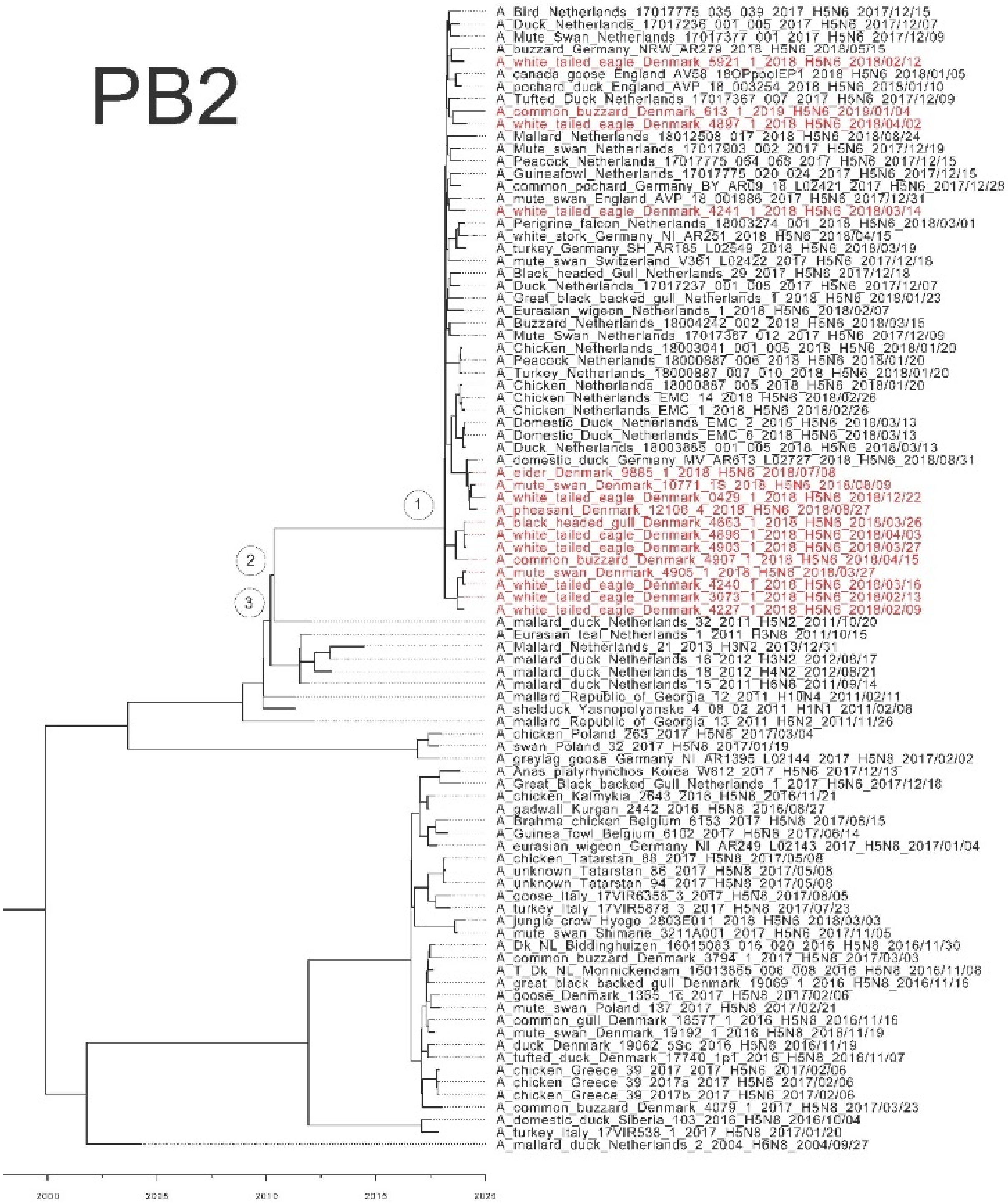

Liang, Y.; Krog, J.S.; Ryt-Hansen, P.; Pedersen, A.G.; Kvisgaard, L.K.; Holm, E.; Nielsen, P.D.; Hammer, A.S.; Madsen, J.J.; Thorup, K.; Larsen, L.E.; Hjulsager, C.K. Molecular Characterization of Highly Pathogenic Avian Influenza Viruses H5N6 Detected in Denmark in 2018–2019. *Viruses* **2021**

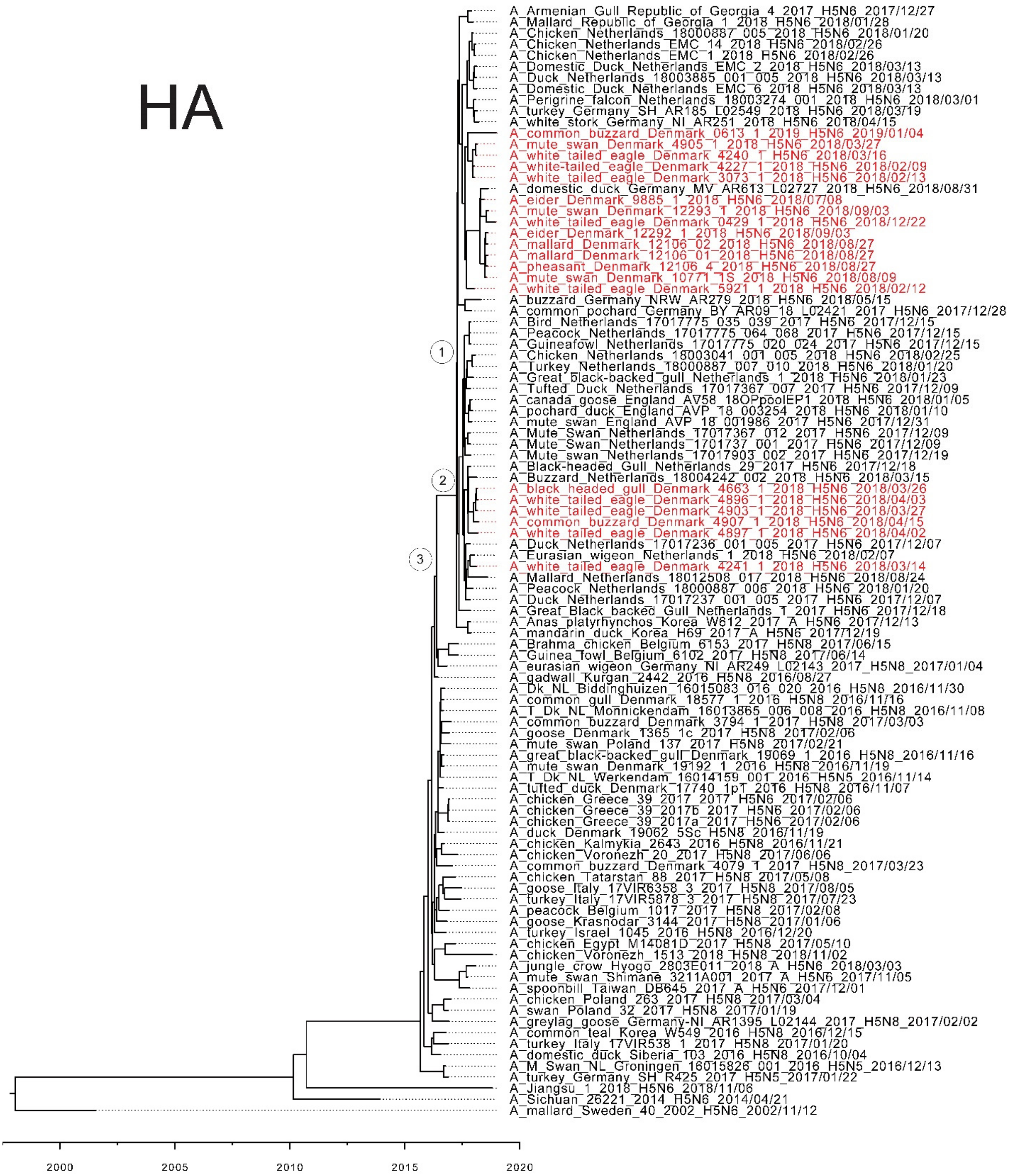
HA
1
2
3
A_Armenian_Gull_Republic_of_Georgia_4_2017_H5N6_2017/12/27
A_Mallard_Republic_of_Georgia_1_2018_H5N6_2018/01/28
A_Chicken_Netherlands_18000887_005_2018_H5N6_2018/01/20
A_Chicken_Netherlands_EMC_14_2018_H5N6_2018/02/26
A_Chicken_Netherlands_EMC_1_2018_H5N6_2018/02/26
A_Domestic_Duck_Netherlands_EMC_2_2018_H5N6_2018/03/13
A_Duck_Netherlands_18003885_001_005_2018_H5N6_2018/03/13
A_Domestic_Duck_Netherlands_EMC_6_2018_H5N6_2018/03/13
A_Perigrine_falcon_Netherlands_18003274_001_2018_H5N6_2018/03/01
A_turkey_Germany_SH_AR185_L02549_2018_H5N6_2018/03/19
A_white_stork_Germany_NI_AR251_2018_H5N6_2018/04/15
A_common_buzzard_Denmark_0613_1_2019_H5N6_2019/01/04
A_mute_swan_Denmark_4905_1_2018_H5N6_2018/03/27
A_white_tailed_eagle_Denmark_4240_1_H5N6_2018/03/16
A_white-tailed_eagle_Denmark_4227_1_2018_H5N6_2018/02/09
A_white_tailed_eagle_Denmark_3073_1_2018_H5N6_2018/02/13
A_domestic_duck_Germany_MV_AR613_L02727_2018_H5N6_2018/08/31
A_eider_Denmark_9885_1_2018_H5N6_2018/07/08
A_mute_swan_Denmark_12293_1_2018_H5N6_2018/09/03
A_white_tailed_eagle_Denmark_0429_1_2018_H5N6_2018/12/22
A_eider_Denmark_12292_1_2018_H5N6_2018/09/03
A_mallard_Denmark_12106_02_2018_H5N6_2018/08/27
A_mallard_Denmark_12106_01_2018_H5N6_2018/08/27
A_pheasant_Denmark_12106_4_2018_H5N6_2018/08/27
A_mute_swan_Denmark_10771_1S_2018_H5N6_2018/08/09
A_white_tailed_eagle_Denmark_5921_1_2018_H5N6_2018/02/12
A_buzzard_Germany_NRW_AR279_2018_H5N6_2018/05/15
A_common_pochard_Germany_BY_AR09_18_L02421_2017_H5N6_2017/12/28
A_Bird_Netherlands_17017775_035_039_2017_H5N6_2017/12/15
A_Peacock_Netherlands_17017775_064_068_2017_H5N6_2017/12/15
A_Guineafowl_Netherlands_17017775_020_024_2017_H5N6_2017/12/15
A_Chicken_Netherlands_18003041_001_005_2018_H5N6_2018/02/25
A_Turkey_Netherlands_18000887_007_010_2018_H5N6_2018/01/20
A_Great_black-backed_gull_Netherlands_1_2018_H5N6_2018/01/23
A_Tufted_Duck_Netherlands_17017367_007_2017_H5N6_2017/12/09
A_canada_goose_England_AV58_18OPpoolEP1_2018_H5N6_2018/01/05
A_pochard_duck_England_AVP_18_003254_2018_H5N6_2018/01/10
A_mute_swan_England_AVP_18_001986_2017_H5N6_2017/12/31
A_Mute_Swan_Netherlands_17017367_012_2017_H5N6_2017/12/09
A_Mute_Swan_Netherlands_1701737_001_2017_H5N6_2017/12/09
A_Mute_swan_Netherlands_17017903_002_2017_H5N6_2017/12/19
A_Black-headed_Gull_Netherlands_29_2017_H5N6_2017/12/18
A_Buzzard_Netherlands_18004242_002_2018_H5N6_2018/03/15
A_black_headed_gull_Denmark_4663_1_2018_H5N6_2018/03/26
A_white_tailed_eagle_Denmark_4896_1_2018_H5N6_2018/04/03
A_white_tailed_eagle_Denmark_4903_1_2018_H5N6_2018/03/27
A_common_buzzard_Denmark_4907_1_2018_H5N6_2018/04/15
A_white_tailed_eagle_Denmark_4897_1_2018_H5N6_2018/04/02
A_Duck_Netherlands_17017236_001_005_2017_H5N6_2017/12/07
A_Eurasian_wigeon_Netherlands_1_2018_H5N6_2018/02/07
A_white_tailed_eagle_Denmark_4241_1_2018_H5N6_2018/03/14
A_Mallard_Netherlands_18012508_017_2018_H5N6_2018/08/24
A_Peacock_Netherlands_18000887_006_2018_H5N6_2018/01/20
A_Duck_Netherlands_17017237_001_005_2017_H5N6_2017/12/07
A_Great_Black_backed_Gull_Netherlands_1_2017_H5N6_2017/12/18
A_Anas_platyrhynchos_Korea_W612_2017_A_H5N6_2017/12/13
A_mandarin_duck_Korea_H69_2017_A_H5N6_2017/12/19
A_Brahma_chicken_Belgium_6153_2017_H5N8_2017/06/15
A_Guinea_fowl_Belgium_6102_2017_H5N8_2017/06/14
A_eurasian_wigeon_Germany_NI_AR249_L02143_2017_H5N8_2017/01/04
A_gadwall_Kurgan_2442_2016_H5N8_2016/08/27
A_Dk_NL_Biddinghuizen_16015083_016_020_2016_H5N8_2016/11/30
A_common_gull_Denmark_18577_1_2016_H5N8_2016/11/16
A_T_Dk_NL_Monnickendam_16013865_006_008_2016_H5N8_2016/11/08
A_common_buzzard_Denmark_3794_1_2017_H5N8_2017/03/03
A_goose_Denmark_1365_1c_2017_H5N8_2017/02/06
A_mute_swan_Poland_137_2017_H5N8_2017/02/21
A_great_black-backed_gull_Denmark_19069_1_2016_H5N8_2016/11/16
A_mute_swan_Denmark_19192_1_2016_H5N8_2016/11/19
A_T_Dk_NL_Werkendam_16014159_001_2016_H5N5_2016/11/14
A_tufted_duck_Denmark_17740_1pT_2016_H5N8_2016/11/07
A_chicken_Greece_39_2017_2017_H5N6_2017/02/06
A_chicken_Greece_39_2017b_2017_H5N6_2017/02/06
A_chicken_Greece_39_2017a_2017_H5N6_2017/02/06
A_duck_Denmark_19062_5Sc_H5N8_2016/11/19
A_chicken_Kalmykia_2643_2016_H5N8_2016/11/21
A_chicken_Voronezh_20_2017_H5N8_2017/06/06
A_common_buzzard_Denmark_4079_1_2017_H5N8_2017/03/23
A_chicken_Tatarstan_88_2017_H5N8_2017/05/08
A_goose_Italy_17VIR6358_3_2017_H5N8_2017/08/05
A_turkey_Italy_17VIR5878_3_2017_H5N8_2017/07/23
A_peacock_Belgium_1017_2017_H5N8_2017/02/08
A_goose_Krasnodar_3144_2017_H5N8_2017/01/06
A_turkey_Israel_1045_2016_H5N8_2016/12/20
A_chicken_Egypt_M14081D_2017_H5N8_2017/05/10
A_chicken_Voronezh_1513_2018_H5N8_2018/11/02
A_jungle_crow_Hyogo_2803E011_2018_A_H5N6_2018/03/03
A_mute_swan_Shimane_3211A001_2017_A_H5N6_2017/11/05
A_spoonbill_Taiwan_DB645_2017_A_H5N6_2017/12/01
A_chicken_Poland_263_2017_H5N8_2017/03/04
A_swan_Poland_32_2017_H5N8_2017/01/19
A_greylag_goose_Germany-NI_AR1395_L02144_2017_H5N8_2017/02/02
A_common_teal_Korea_W549_2016_H5N8_2016/12/15
A_turkey_Italy_17VIR538_1_2017_H5N8_2017/01/20
A_domestic_duck_Siberia_103_2016_H5N8_2016/10/04
A_M_Swan_NL_Groningen_16015826_001_2016_H5N5_2016/12/13
A_turkey_Germany_SH_R425_2017_H5N5_2017/01/22
A_Jiangsu_1_2018_H5N6_2018/11/06
A_Sichuan_26221_2014_H5N6_2014/04/21
A_mallard_Sweden_40_2002_H5N6_2002/11/12
2000
2005
2010
2015
2020

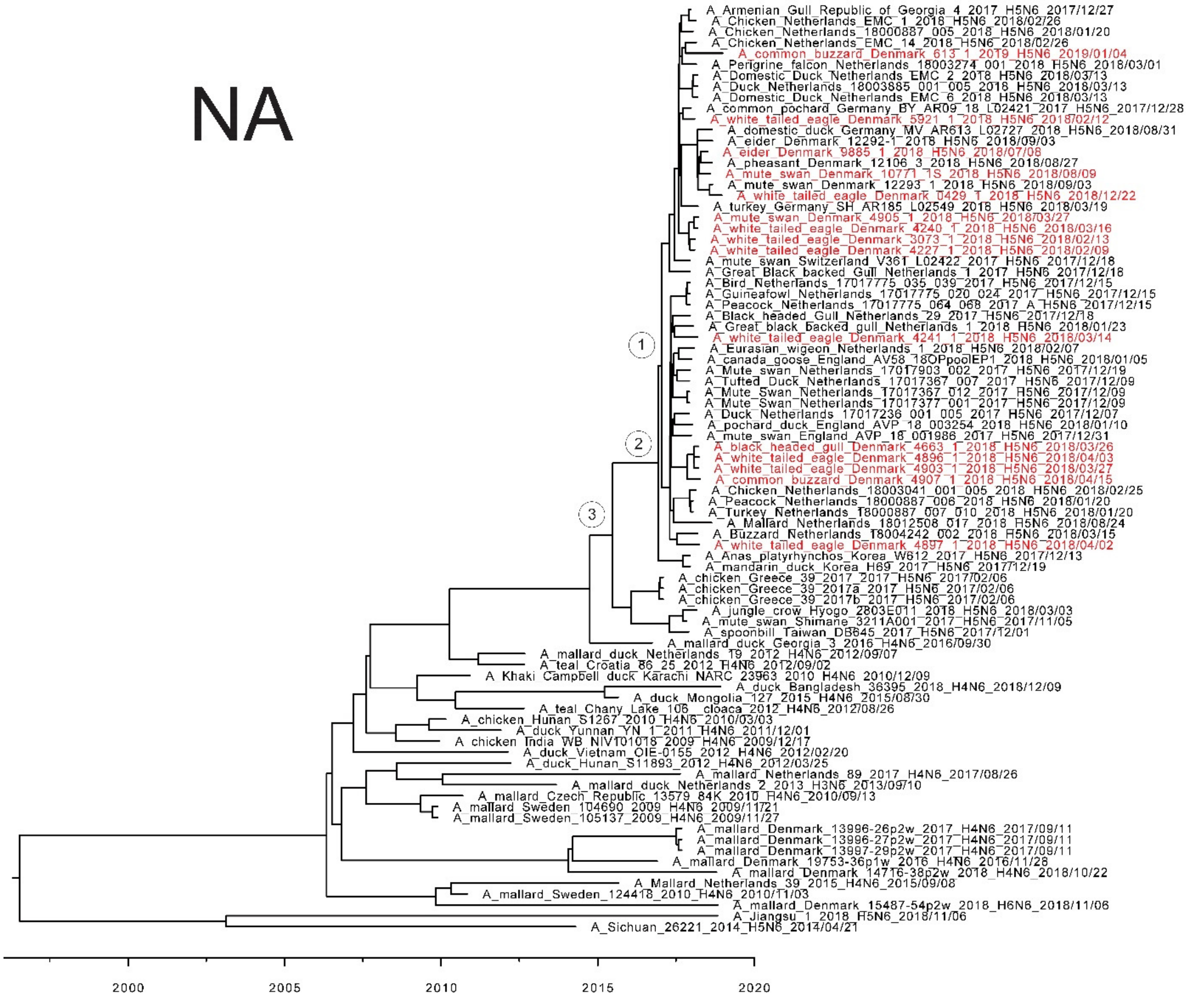
NA
A_Armenian_Gull_Republic_of_Georgia_4_2017_H5N6_2017/12/27
A_Chicken_Netherlands_EMC_1_2018_H5N6_2018/02/26
A_Chicken_Netherlands_18000887_005_2018_H5N6_2018/01/20
A_Chicken_Netherlands_EMC_14_2018_H5N6_2018/02/26
A_common_buzzard_Denmark_613_1_2019_H5N6_2019/01/04
A_Perigrine_falcon_Netherlands_18003274_001_2018_H5N6_2018/03/01
A_Domestic_Duck_Netherlands_EMC_2_2018_H5N6_2018/03/13
A_Duck_Netherlands_18003885_001_005_2018_H5N6_2018/03/13
A_Domestic_Duck_Netherlands_EMC_6_2018_H5N6_2018/03/13
A_common_pochard_Germany_BY_AR09_18_L02421_2017_H5N6_2017/12/28
A_white_tailed_eagle_Denmark_5921_1_2018_H5N6_2018/02/12
A_domestic_duck_Germany_MV_AR613_L02727_2018_H5N6_2018/08/31
A_eider_Denmark_12292-1_2018_H5N6_2018/09/03
A_eider_Denmark_9885_1_2018_H5N6_2018/07/08
A_pheasant_Denmark_12106_3_2018_H5N6_2018/08/27
A_mute_swan_Denmark_10771_1S_2018_H5N6_2018/08/09
A_mute_swan_Denmark_12293_1_2018_H5N6_2018/09/03
A_white_tailed_eagle_Denmark_0429_1_2018_H5N6_2018/12/22
A_turkey_Germany_SH_AR185_L02549_2018_H5N6_2018/03/19
A_mute_swan_Denmark_4905_1_2018_H5N6_2018/03/27
A_white_tailed_eagle_Denmark_4240_1_2018_H5N6_2018/03/16
A_white_tailed_eagle_Denmark_3073_1_2018_H5N6_2018/02/13
A_white_tailed_eagle_Denmark_4227_1_2018_H5N6_2018/02/09
A_mute_swan_Switzerland_V361_L02422_2017_H5N6_2017/12/18
A_Great_Black_backed_Gull_Netherlands_1_2017_H5N6_2017/12/18
A_Bird_Netherlands_17017775_035_039_2017_H5N6_2017/12/15
A_Guineafowl_Netherlands_17017775_020_024_2017_H5N6_2017/12/15
A_Peacock_Netherlands_17017775_064_068_2017_A_H5N6_2017/12/15
A_Black_headed_Gull_Netherlands_29_2017_H5N6_2017/12/18
A_Great_black_backed_gull_Netherlands_1_2018_H5N6_2018/01/23
A_white_tailed_eagle_Denmark_4241_1_2018_H5N6_2018/03/14
A_Eurasian_wigeon_Netherlands_1_2018_H5N6_2018/02/07
A_canada_goose_England_AV58_18OPpoolEP1_2018_H5N6_2018/01/05
A_Mute_swan_Netherlands_17017903_002_2017_H5N6_2017/12/19
A_Tufted_Duck_Netherlands_17017367_007_2017_H5N6_2017/12/09
A_Mute_Swan_Netherlands_17017367_012_2017_H5N6_2017/12/09
A_Mute_Swan_Netherlands_17017377_001_2017_H5N6_2017/12/09
A_Duck_Netherlands_17017236_001_005_2017_H5N6_2017/12/07
A_pochard_duck_England_AVP_18_003254_2018_H5N6_2018/01/10
A_mute_swan_England_AVP_18_001986_2017_H5N6_2017/12/31
A_black_headed_gull_Denmark_4663_1_2018_H5N6_2018/03/26
A_white_tailed_eagle_Denmark_4896_1_2018_H5N6_2018/04/03
A_white_tailed_eagle_Denmark_4903_1_2018_H5N6_2018/03/27
A_common_buzzard_Denmark_4907_1_2018_H5N6_2018/04/15
A_Chicken_Netherlands_18003041_001_005_2018_H5N6_2018/02/25
A_Peacock_Netherlands_18000887_006_2018_H5N6_2018/01/20
A_Turkey_Netherlands_18000887_007_010_2018_H5N6_2018/01/20
A_Mallard_Netherlands_18012508_017_2018_H5N6_2018/08/24
A_Buzzard_Netherlands_18004242_002_2018_H5N6_2018/03/15
A_white_tailed_eagle_Denmark_4897_1_2018_H5N6_2018/04/02
A_Anas_platyrhynchos_Korea_W612_2017_H5N6_2017/12/13
A_mandarin_duck_Korea_H69_2017_H5N6_2017/12/19
A_chicken_Greece_39_2017_2017_H5N6_2017/02/06
A_chicken_Greece_39_2017a_2017_H5N6_2017/02/06
A_chicken_Greece_39_2017b_2017_H5N6_2017/02/06
A_jungle_crow_Hyogo_2803E011_2018_H5N6_2018/03/03
A_mute_swan_Shimane_3211A001_2017_H5N6_2017/11/05
A_spoonbill_Taiwan_DB645_2017_H5N6_2017/12/01
A_mallard_duck_Georgia_3_2016_H4N6_2016/09/30
A_mallard_duck_Netherlands_19_2012_H4N6_2012/09/07
A_teal_Croatia_86_25_2012_H4N6_2012/09/02
A_Khaki_Campbell_duck_Karachi_NARC_23963_2010_H4N6_2010/12/09
A_duck_Bangladesh_36395_2018_H4N6_2018/12/09
A_duck_Mongolia_127_2015_H4N6_2015/08/30
A_teal_Chany_Lake_106__cloaca_2012_H4N6_2012/08/26
A_chicken_Hunan_S1267_2010_H4N6_2010/03/03
A_duck_Yunnan_YN_1_2011_H4N6_2011/12/01
A_chicken_India_WB_NIV101018_2009_H4N6_2009/12/17
A_duck_Vietnam_OIE-0155_2012_H4N6_2012/02/20
A_duck_Hunan_S11893_2012_H4N6_2012/03/25
A_mallard_Netherlands_89_2017_H4N6_2017/08/26
A_mallard_duck_Netherlands_2_2013_H3N6_2013/09/10
A_mallard_Czech_Republic_13579_84K_2010_H4N6_2010/09/13
A_mallard_Sweden_104690_2009_H4N6_2009/11/21
A_mallard_Sweden_105137_2009_H4N6_2009/11/27
A_mallard_Denmark_13996-26p2w_2017_H4N6_2017/09/11
A_mallard_Denmark_13996-27p2w_2017_H4N6_2017/09/11
A_mallard_Denmark_13997-29p2w_2017_H4N6_2017/09/11
A_mallard_Denmark_19753-36p1w_2016_H4N6_2016/11/28
A_mallard_Denmark_14716-38p2w_2018_H4N6_2018/10/22
A_Mallard_Netherlands_39_2015_H4N6_2015/09/08
A_mallard_Sweden_124418_2010_H4N6_2010/11/03
A_mallard_Denmark_15487-54p2w_2018_H6N6_2018/11/06
A_Jiangsu_1_2018_H5N6_2018/11/06
A_Sichuan_26221_2014_H5N6_2014/04/21
1
2
3
2000
2005
2010
2015
2020

Table S1. Results from active surveillance for avian influenza virus in clinically healthy wild bird, Denmark, 2018

Sampling time, 2018 quarter	Location	Species	No. Birds	No. Pools*	Avian influenza virus positive pools	H5/H7 positive pools
1	Copenhagen	Eurasian coot	5	1	0	0
1	Copenhagen	Common moorhen	1	1	0	0
1	Copenhagen	Mallard	5	1	0	0
1	Copenhagen	Black-headed gull	1	1	0	0
1	Copenhagen	Common gull	1	1	0	0
3	Jutland	Mallard	16	6	2	0
3	Jutland	Eurasian teal	42	13	8	0
3	Jutland	Eurasian wigeon	1	1	0	0
3	Jutland	Northern shoveler	3	2	1	0
3	Jutland	Northern pintail	2	2	0	0
3	Jutland	Mallard	50	10	5	0
3	Jutland	Greylag goose	1	1	0	0
3	Jutland	Common gull	2	2	0	0
3	Zealand	Mallard	13	3	0	0
3	Zealand	Gadwall	1	1	0	0
3	Zealand	Eurasian teal	2	1	1	0
3	Zealand	Eurasian sparrowhawk	8	4	0	0
3	Zealand	Common kestrel	1	1	0	0
3	Zealand	Mallard	40	8	6	1xH5 LPAI
4	Funen	Eurasian coot	9	3	1	0
4	Funen	Common moorhen	4	2	0	0
4	Funen	Mallard	42	10	0	0
4	Funen	Black-headed gull	3	2	0	0
4	Funen	Mute swan	7	3	0	0
4	Funen	Mallard	69	17	4	0
4	Jutland	Barnacle goose	79	17	0	0
4	Jutland	Common moorhen	1	1	0	0
4	Jutland	Mallard	19	8	3	0
4	Jutland	Black-headed gull	3	1	0	0
4	Jutland	Eurasian teal	95	24	11	0
4	Jutland	Eurasian wigeon	14	5	0	0
4	Jutland	Eurasian woodcock	8	3	0	0
4	Jutland	Northern pintail	5	4	1	0
4	Jutland	Mallard	188	38	5	0
4	Jutland	Hooded crow	1	1	0	0
4	Jutland	Eurasian teal	12	3	0	0
4	Jutland	Common buzzard	1	1	0	0
4	Jutland	Whooper swan	4	4	0	0
4	Jutland	Common gull	1	1	0	0
4	Copenhagen	Common gull	1	1	0	0
4	Zealand	Western jackdaw	5	1	0	0
4	Zealand	Barnacle goose	19	5	0	0
4	Zealand	Pheasant	10	2	0	0
4	Zealand	Rough-legged buzzard	1	1	0	0
4	Zealand	Greylag goose	11	3	0	0
4	Zealand	Mute swan	2	1	0	0
4	Zealand	Eurasian teal	2	2	0	0
4	Zealand	Eurasian wigeon	2	1	0	0
4	Zealand	Common wood pigeon	3	1	0	0
4	Zealand	Eurasian sparrowhawk	12	11	1	0
4	Zealand	European herring gull	1	1	0	0
4	Zealand	Common kestrel	2	2	0	0
4	Zealand	Mallard	35	11	8	0
Total			866	251	57	1

*Wild birds were tested for avian influenza virus in pools of cloacal swab samples from up to five birds of the same species, sampled at the same time and location.

Table S2. Results from active surveillance for avian influenza virus in clinically healthy wild bird, Denmark, 2019

Sampling time, 2019 quarter	Location	Species	No. birds	No. Pools*	Avian influenza virus positive pools	H5/H7 positive pools
1	Copenhagen	Eurasian Coot	1	1	0	0
1	Copenhagen	Mallard	4	1	0	0
1	Copenhagen	Tufted duck	4	1	0	0
3	Funen	Mute swan	1	1	0	0
3	Funen	Peregrine falcon	1	1	0	0
3	Jutland	Mallard	102	21	16	0
3	Jutland	Mallard	7	2	1	1xH5 LPAI
3	Jutland	Eurasian teal	34	9	7	0
3	Jutland	Eurasian wigeo	5	2	1	0
3	Jutland	Northern shoveler	4	2	1	1xH5 LPAI
3	Zealand	Mallard	50	12	7	0
3	Zealand	Merlin	1	1	1	0
3	Zealand	Eurasian sparrowhawk	12	8	0	0
4	Funen	Pheasant	40	8	0	0
4	Funen	Mallard	100	24	5	0
4	Funen	Eurasian coot	1	1	0	0
4	Funen	Mallard	6	3	1	0
4	Funen	Black-headed gull	7	2	0	0
4	Funen	Mute swan	6	3	0	0
4	Funen	Eurasian teal	2	2	1	0
4	Jutland	Mallard	48	10	2	0
4	Jutland	Mallard	24	9	2	0
4	Jutland	Gadwall	1	1	0	0
4	Jutland	Eurasian teal	30	9	5	1xH5 LPAI
4	Jutland	Eurasian wigeon	68	17	10	1xH5 LPAI
4	Jutland	Eurasian woodcock	2	1	0	0
4	Jutland	Northern pintail	3	3	0	0
4	Jutland	Common gull	48	10	0	0
4	Copenhagen	Greylag goose	35	7	0	0
4	Zealand	Pheasant	80	16	0	0
4	Zealand	Mallard	50	11	6	0
4	Zealand	Barnacle goose	48	12	1	0
4	Zealand	Mallard	2	1	0	0
4	Zealand	Short-eared owl	1	1	0	0
4	Zealand	Common buzzard	1	1	0	0
4	Zealand	Eurasian wigeon	2	1	0	0
4	Zealand	Rook	8	2	0	0
4	Zealand	Northern pintail	1	1	0	0
4	Zealand	Eurasian sparrowhawk	19	12	2	0
4	Zealand	Common ringed plover	1	1	0	0
4	Zealand	Common gull	15	3	0	0
4	Zealand	European herring gull	17	4	1	0
Total			892	238	70	4

*Wild birds were tested for avian influenza virus in pools of cloacal swab samples from up to five birds of the same species, sampled at the same time and location.

Table S3: Results of avian influenza virus wild bird passive surveillance, Denmark, 2018

Sampling time	Location	Species	Avian influenza virus result
01-02-2018	Thisted	Common buzzard	Negative
09-02-2018	Næstved	White-tailed eagle	HPAI H5N6 detected
13-02-2018	Slagelse	White-tailed eagle	HPAI H5N6 detected
12-02-2018	Guldborgsund	White-tailed eagle	HPAI H5N6 detected
18-02-2018	Greve	Common buzzard	Negative
26-02-2018	Slagelse	Common buzzard	Negative
28-02-2018	Hillerød	Common buzzard	Negative
02-03-2018	Lyngby-Taarbæk	Common buzzard	Negative
02-03-2018	Herning	Common buzzard	Negative
02-03-2018	Holbæk	Common buzzard	Negative
03-03-2018	Slagelse	Eurasian bittern	Negative
03-03-2018	Tønder	Grey heron	Negative
06-03-2018	Slagelse	Grey heron	Negative
06-03-2018	Thisted	Eurasian eagle-owl	Negative
08-03-2018	Faaborg-Midtfyn	Common buzzard	Negative
08-03-2018	Syddjurs	Common buzzard	Negative
08-03-2018	Haderslev	Common buzzard	Negative
10-03-2018	Vordingborg	Common kestrel	Negative
10-03-2018	Thisted	White-tailed eagle	HPAI H5N6 detected
12-03-2018	Aabenraa	Common buzzard	Negative
14-03-2018	Thisted	White-tailed eagle	HPAI H5N6 detected
15-03-2018	Fredensborg	Common buzzard	Negative
15-03-2018	Guldborgsund	Mute swan	Negative
15-03-2018	Lolland	Grey heron	Negative
15-03-2018	Stevns	Common buzzard	Negative
15-03-2018	Guldborgsund	Common buzzard	Negative
16-03-2018	Svendborg	Common buzzard	Negative
16-03-2018	Lolland	White-tailed eagle	HPAI H5N6 detected
17-03-2018	Faxe	Common buzzard	Negative
18-03-2018	Silkeborg	Common buzzard	Negative
19-03-2018	Vejle	Common buzzard	Negative
20-03-2018	Aalborg	Common buzzard	Negative
20-03-2018	Aalborg	Common buzzard	Negative
20-03-2018	Stevns	Common buzzard	Negative
21-03-2018	Gribskov	Common buzzard	Negative
21-03-2018	Syddjurs	Common buzzard	Negative
22-03-2018	Helsingør	Grey heron	Negative
22-03-2018	Svendborg	Common buzzard	Negative
22-03-2018	Odense	Common buzzard	Negative
24-03-2018	Mariagerfjord	Common buzzard	Negative
24-03-2018	Faaborg-Midtfyn	Common buzzard	Negative
24-03-2018	Køge	Common kestrel	Negative
24-03-2018	Bornholm	Common buzzard	HPAI H5N6 detected
24-03-2018	Aalborg	Common buzzard	HPAI H5N6 detected
25-03-2018	Aalborg	Hooded crow	HPAI H5N6 detected
25-03-2018	Aalborg	Common buzzard	HPAI H5N6 detected
25-03-2018	Vordingborg	Common buzzard	Negative
26-03-2018	Lolland	Great cormorant	Negative
26-03-2018	Kolding	Common buzzard	Negative
26-03-2018	Helsingør	Grey heron	Negative
26-03-2018	Hedensted	Common buzzard	Negative
26-03-2018	Slagelse	Black-headed gull	HPAI H5N6 detected
26-03-2018	Holbæk	European herring gull	Negative
26-03-2018	Aalborg	Mute swan	Negative
27-03-2018	Sorø	Common buzzard	Negative
27-03-2018	Vejle	Common buzzard	Negative
27-03-2018	Lejre	White-tailed eagle	HPAI H5N6 detected
27-03-2018	Slagelse	White-tailed eagle	HPAI H5N6 detected
27-03-2018	Næstved	White-tailed eagle	HPAI H5N6 detected
27-03-2018	Slagelse	Great cormorant	HPAI H5N6 detected
27-03-2018	Slagelse	European herring gull	HPAI H5N6 detected
27-03-2018	Guldborgsund	Mute swan	HPAI H5N6 detected
28-03-2018	Aalborg	Hooded crow	HPAI H5N6 detected

28-03-2018	Slagelse	Common buzzard	Negative
01-04-2018	Tårnby	Common buzzard	HPAI H5N6 detected
01-04-2018	Silkeborg	Common buzzard	Negative
02-04-2018	Skanderborg	Common buzzard	Negative
02-04-2018	Kalundborg	Common buzzard	HPAI H5N6 detected
02-04-2018	Kalundborg	Common buzzard	HPAI H5N6 detected
02-04-2018	Slagelse	White-tailed eagle	HPAI H5N6 detected
03-04-2018	Slagelse	Common buzzard	Negative
03-04-2018	Slagelse	White-tailed eagle	HPAI H5N6 detected
05-04-2018	Favrskov	Tawny owl	Negative
08-04-2018	Jammerbugt	Common buzzard	HPAI H5N6 detected
09-04-2018	Favrskov	Common buzzard	HPAI H5N6 detected
11-04-2018	Faxe	Common buzzard	HPAI H5N6 detected
11-04-2018	Faaborg-Midtfyn	Mute swan	HPAI H5N6 detected
12-04-2018	Kerteminde	White-tailed eagle	Negative
13-04-2018	Faaborg-Midtfyn	Common buzzard	Negative
15-04-2018	Sønderborg	Common buzzard	HPAI H5N6 detected
15-04-2018	Aarhus	Common buzzard	HPAI H5N6 detected
18-04-2018	Tønder	Black-headed gull	Negative
24-04-2018	Ringsted	Greylag goose	Negative
09-05-2018	Esbjerg	Common eider	Negative
22-05-2018	Tårnby	Mute swan	Negative
28-05-2018	Kalundborg	Common eider	Negative
03-07-2018	Guldborgsund	Mallard	Negative
05-07-2018	Gribskov	Common buzzard	Negative
08-07-2018	Lolland	Common eider	HPAI H5N6 detected
03-08-2018	Kalundborg	Common pheasant	Negative
03-08-2018	Kalundborg	Common pheasant	Negative
03-08-2018	Kalundborg	Common pheasant	Negative
03-08-2018	Kalundborg	Common pheasant	Negative
03-08-2018	Kalundborg	Common pheasant	Negative
07-08-2018	Guldborgsund	Mute swan	Negative
09-08-2018	Slagelse	Mute swan	HPAI H5N6 detected
15-08-2018	Odsherred	Great cormorant	Negative
25-08-2018	Guldborgsund	Mute swan	AIV detected, not H5/H7
25-08-2018	Guldborgsund	Mute swan	AIV detected, not H5/H7
25-08-2018	Guldborgsund	Mute swan	AIV detected, not H5/H7
25-08-2018	Guldborgsund	Mute swan	AIV detected, not H5/H7
27-08-2018	Faxe	Greylag goose	Negative
27-08-2018	Slagelse	Mallard	HPAI H5N6
27-08-2018	Slagelse	Mallard	HPAI H5N6
27-08-2018	Slagelse	Common pheasant	HPAI H5N6
27-08-2018	Slagelse	Common pheasant	HPAI H5N6
02-09-2018	Middelfart	Mute swan	Negative
03-09-2018	Lolland	Greylag goose	HPAI H5N6 detected
03-09-2018	Guldborgsund	Mute swan	HPAI H5N6 detected
03-09-2018	Guldborgsund	Common eider	HPAI H5N6 detected
07-09-2018	Lolland	Mute swan	Negative
10-09-2018	Varde	European herring gull	Negative
23-08-2018	Vejle	Osprey	Negative
06-09-2018	Silkeborg	White-tailed eagle	Negative
15-09-2018	Vejle	Grey heron	Negative
19-09-2018	Slagelse	Common pheasant	HPAI H5N6 detected
19-09-2018	Slagelse	Common pheasant	HPAI H5N6 detected
19-09-2018	Slagelse	Common pheasant	HPAI H5N6 detected
24-09-2018	Ringkøbing-Skjern	Barn owl	Negative
24-09-2018	Faxe	European herring gull	Negative
26-09-2018	Dragør	Common kestrel	Negative
05-10-2018	Køge	European herring gull	Negative
15-10-2018	Vejen	Grey heron	Negative
15-10-2018	Skive	Great cormorant	Negative
15-10-2018	Skive	Great cormorant	Negative
15-10-2018	Skive	Great cormorant	Negative
15-10-2018	Skive	Great cormorant	Negative
15-10-2018	København	Eurasian magpie	Negative
21-10-2018	Horsens	Common kestrel	Negative

24-10-2018	Guldborgsund	Mute swan	Negative
26-10-2018	Sorø	Common buzzard	Negative
30-10-2018	Holbæk	European herring gull	Negative
05-11-2018	Tønder	Common buzzard	Negative
12-11-2018	Slagelse	Barnacle goose	Negative
12-11-2018	Slagelse	Barnacle goose	Negative
12-11-2018	Slagelse	Barnacle goose	Negative
12-11-2018	Slagelse	Barnacle goose	Negative
17-11-2018	Odense	Eurasian woodcock	Negative
25-11-2018	Haderslev	Common buzzard	Negative
17-12-2018	Sønderborg	Mallard	Negative
17-12-2018	Holbæk	Common buzzard	Negative
17-12-2018	Assens	Common buzzard	Negative
20-12-2018	Vordingborg	Mute swan	Negative
20-11-2018	Aalborg	Golden eagle	Negative
22-12-2018	Næstved		HPAI H5N6 detected
23-11-2018	Guldborgsund	Common buzzard	Negative
	Total		42 HPAI H5N6

Table S4. Results of avian influenza virus wild bird passive surveillance, Denmark, 2019.

Sampling time	Location	Species	Avian influenza virus result
01-01-2019	Sønderborg	Common buzzard	Negative
01-01-2019	Sønderborg	Red kite	Negative
01-01-2019	Næstved	Mute swan	Negative
03-01-2019	Faxe	Common kestrel	Negative
04-01-2019	Odsherred	Common buzzard	HPAI H5N6 detected
15-01-2019	Køge	Peregrine falcon	Negative
16-01-2019	Gribskov	Common buzzard	Negative
17-01-2019	Holbæk	Common buzzard	Negative
20-01-2019	Esbjerg	Common buzzard	Negative
20-01-2019	Guldborgsund	Common buzzard	Negative
21-01-2019	Ringsted	Hen harrier	Negative
22-01-2019	Jammerbugt	Common murre	Negative
22-01-2019	Jammerbugt	Common murre	Negative
22-01-2019	Billund	Whooper swan	Negative
22-01-2019	Jammerbugt	Razorbill	Negative
23-01-2019	Guldborgsund	Common buzzard	Negative
24-01-2019	Guldborgsund	Mute swan	Negative
26-01-2019	Lolland	Rough-legged buzzard	Negative
28-01-2019	Langeland	Great cormorant	Negative
28-01-2019	Langeland	Great cormorant	Negative
28-01-2019	Langeland	Great cormorant	Negative
28-01-2019	Rebild	Common buzzard	Negative
30-01-2019	Slagelse	Common buzzard	Negative
30-01-2019	Odsherred	Common buzzard	Negative
03-02-2019	Sorø	Common buzzard	Negative
04-02-2019	Guldborgsund	Peregrine falcon	Negative
05-02-2019	Roskilde	Common buzzard	Negative
11-02-2019	Vordingborg	Northern goshawk	Negative
11-02-2019	Guldborgsund	Common buzzard	Negative
12-03-2019	Guldborgsund	Red kite	Negative
15-02-2019	Lolland	White-tailed eagle	Negative
15-02-2019	Herning	Common buzzard	Negative
16-02-2019	Silkeborg	Whooper swan	AIV detected, not H5/H7
16-02-2019	Silkeborg	Whooper swan	Negative
18-02-2019	Ringkøbing-Skjern	Common buzzard	Negative
18-02-2019	Silkeborg	Common buzzard	Negative
20-02-2019	Faxe	Common gull	Negative
20-02-2019	Faxe	Hooded crow	Negative
21-02-2019	Faaborg-Midtfyn	Great cormorant	Negative
21-02-2019	Hørsholm	Mute swan	Negative
23-02-2019	Sorø	Common buzzard	Negative
23-02-2019	Sorø	Common buzzard	Negative
28-02-2019	Nordfyns	Common buzzard	Negative
28-02-2019	Nordfyns	Common buzzard	Negative
02-03-2019	Vesthimmerlands	Eurasian sparrowhawk	Negative
10-03-2019	Varde	Common buzzard	Negative
11-03-2019	Egedal	Eurasian sparrowhawk	Negative
12-03-2019	Helsingør	European herring gull	Negative
12-03-2019	Helsingør	European herring gull	Negative
18-03-2019	Lolland	Mute swan	Negative
19-03-2019	Holbæk	Common buzzard	Negative
24-03-2019	Mariagerfjord	Eurasian magpie	Negative
24-03-2019	Mariagerfjord	Eurasian sparrowhawk	Negative
27-03-2019	Lolland	Great cormorant	Negative
01-04-2019	Ringkøbing-Skjern	Mute swan	Negative
15-04-2019	Ringsted	Common buzzard	Negative
15-04-2019	Kolding	Black-headed gull	Negative
15-04-2019	Kolding	Gulls, terns	Negative
20-04-2019	Kolding	Common buzzard	Negative
20-04-2019	Kolding	Common buzzard	Negative
24-04-2019	Vordingborg	White-tailed eagle	Negative
30-04-2019	Holbæk	Common buzzard	Negative
25-05-2019	Randers	Common buzzard	Negative

27-05-2019	Gentofte	Eurasian coot	Negative
27-05-2019	Guldborgsund	Common buzzard	Negative
10-06-2019	Syddjurs	Common buzzard	Negative
12-06-2019	Ikast-Brande	Western jakdaw	Negative
11-07-2019	Jammerbugt	European herring gull	Negative
11-07-2019	Jammerbugt	European herring gull	Negative
11-07-2019	Jammerbugt	European herring gull	Negative
09-07-2019	Vordingborg	European herring gull	Negative
09-07-2019	Vordingborg	European herring gull	Negative
09-07-2019	Vordingborg	European herring gull	Negative
09-07-2019	Vordingborg	European herring gull	AIV detected, not H5/H7
09-07-2019	Vordingborg	European herring gull	Negative
09-07-2019	Vordingborg	European herring gull	Negative
09-07-2019	Vordingborg	European herring gull	Negative
23-07-2019	Solrød	Eurasian magpie	Negative
31-07-2019	København	Mallard	Negative
31-07-2019	København	Mallard	Negative
31-07-2019	Rødovre	Eurasian coot	Negative
31-07-2019	Rødovre	Eurasian coot	Negative
31-07-2019	København	Black-headed gull	Negative
31-07-2019	København	Mallard	Negative
05-08-2019	Holbæk	Mallard	Negative
05-08-2019	Holbæk	Mallard	Negative
06-08-2019	Viborg	Mute swan	Negative
23-08-2019	Holbæk	European honey buzzard	Negative
29-08-2019	København	European herring gull	Negative
31-08-2019	Sønderborg	Mute swan	Negative
03-09-2019	København	Eurasian teal	Negative
05-09-2019	Hvidovre	Mallard	Negative
07-09-2019	Svendborg	White-tailed eagle	Negative
15-09-2019	Frederikshavn	European herring gull	Negative
24-09-2019	Herning	Rook	Negative
25-09-2019	Aarhus	Great cormorant	Negative
02-10-2019	Haderslev	Eurasian sparrowhawk	Negative
14-10-2019	Skive	Black-headed gull	Negative
03-11-2019	Næstved	Red kite	Negative
09-11-2019	Fredensborg	Northern goshawk	Negative
17-11-2019	Aalborg	Eurasian coot	Negative
18-11-2019	Kolding	Barn owl	Negative
18-11-2019	Kolding	Barn owl	Negative
21-11-2019	Næstved	Common buzzard	Negative
22-11-2019	Tønder	Northern goshawk	Negative
07-12-2019	Struer	Black-headed gull	Negative
10-12-2019	København	Great cormorant	Negative
	Total		1 HPAI H5N6

Table S5: Details of the Danish clade 2.3.4.4b H5N6 HPAIV that have been sequenced in this study

Virus	Subtype	Sampling location in Denmark	Collection date	Material	GenBank accession no.
A/white-tailed eagle/Denmark/4227-01/2018	H5N6	Næstved	09/02/2018	Primary material	MT781548 - MT781554
A/white-tailed eagle/Denmark/5921-01/2018	H5N6	Guldborgsund	12/02/2018	Primary material	MT781596 - MT781603
A/white-tailed eagle/Denmark/3073-01/2018	H5N6	Slagelse	13/02/2018	Primary material	MT781540 - MT781547
A/white-tailed eagle/Denmark/4241-01/2018	H5N6	Thisted	10/03/2018	Primary material	MT781564 - MT781571
A/white-tailed eagle/Denmark/4240-01/2018	H5N6	Lolland	16/03/2018	Primary material	MT781556 - MT781563
A/black-headed gull/Denmark/4663-01/2018	H5N6	Slagelse	26/03/2018	Primary material	MT781476 - MT781483
A/mute swan/Denmark/4905-01/2018	H5N6	Guldborgsund	27/03/2018	Primary material	MT781516 - MT781523
A/white-tailed eagle/Denmark/4903-01/2018	H5N6	Slagelse	27/03/2018	Primary material	MT781588 - MT781596
A/white-tailed eagle/Denmark/4897-01/2018	H5N6	Slagelse	02/04/2018	Primary material	MT781580 - MT781587
A/white-tailed eagle/Denmark/4896-01/2018	H5N6	Slagelse	03/04/2018	Isolate	MT781572 - MT781579
A/common buzzard/Denmark/4907/2018	H5N6	Sønderborg	15/04/2018	Primary material	MT781484 - MT781491
A/eider/Denmark/9885-01/1/2018	H5N6	Lolland	08/07/2018	Primary material	MT781500 - MT781507
A/mute swan/Denmark/10771-01S/2018	H5N6	Slagelse	09/08/2018	Primary material	MT781508 - MT781515
A/mallard/Denmark/12106-01/2018	H5N6	Slagelse	27/08/2018	Primary material	MT781472
A/mallard/Denmark/12106-02/2018	H5N6	Slagelse	27/08/2018	Primary material	MT781473
A/pheasant/Denmark/12106-03/2018	H5N6	Slagelse	27/08/2018	Primary material	MT781524 - MT781531
A/mute swan/Denmark/12293-1/2018	H5N6	Guldborgsund	03/09/2018	Primary material	MT781474 - MT781475
A/eider/Denmark/12292-1/2018	H5N6	Guldborgsund	03/09/2018	Primary material	MT781470 - MT781471
A/white-tailed eagle/Denmark/0429-01/2018	H5N6	Næstved	22/12/2018	Primary material	MT781532 - MT781539
A/common buzzard/Denmark/0613-01/2019	H5N6	Odsherred	04/01/2019	Primary material	MT781492 - MT781499

Table S6: Risk signatures. Based on Beerens, N.; Koch, G.; Heutink, R.; Harders, F.; Vries, D.P.E.; Ho, C.; Bossers, A.; Elbers, A. Novel highly pathogenic avian influenza A(H5N6) virus in the Netherlands, december 2017. *Emerg. Infect. Dis.* **2018**, *24*, 770–773, doi:10.3201/eid2404.172124.

Protein	Position	Present	Increased
PB2	D265G	No	Replication
	Q591K	No	Replication
	E627K	No	Virulence
	D701N	No	Replication
	S714R	No	Replication
PB1	V3A	No	Virulence
	H99Y	No	Airborne transmission
	K207R	No	Virulence
	N328K	No	Virulence
	I368V	No	Airborne transmission
	N375S	No	Virulence
PA	T85I	No	Replication
	T97I	No	Virulence
	G186M	No	Replication
	L336S	No	Replication
	S409N	No	Human transmission
	K615N	No	Replication
	G631S	No	Virulence
H5	Various residues	No	Binding to a2,6
	119Y, 172A, 238L, 240S	Only 172A	Airborne transmission
	450K, 459K, 403I	No	Acid stability
NP	R99K	No	Airborne transmission
	N391K	No	Replication
	S345N	No	Airborne transmission
	Q357K	No	Virulence
N6	V116A	No	Resistance to oseltamivir, zanamivir
	E119V	No	Resistance to oseltamivir, zanamivir
	Q136K	No	Resistance to zanamivir
	D199N	Yes	Resistance to oseltamivir
M1	N30D	Yes*	Virulence
	T139A	No	Virulence
	Y215A	Yes*	Virulence
NS1	P42S	Yes*	Virulence
	D92E	No	Virulence
	L103F	Yes*	Virulence
	I106M	Yes*	Virulence
	80-04	No	Virulence

*Features of H5 clade 2.3.4.4 viruses.

Table S7: Acknowledgement of laboratories providing sequences to the GISAID EpiFlu database and NCBI Genbank database.

GISAID Isolate ID/ Genbank Accession no.	Country	Isolate Name	Subtype	Collection Date	Submitting Laboratory
EPI_ISL_332433	The Netherlands	A/Bird/Netherlands/17017775-035-039/2017	H5N6	15/12/2017	Wageningen Bioveterinary Research
EPI_ISL_332434	The Netherlands	A/Peacock/Netherlands/17017775-064-068/2017	H5N6	15/12/2017	Wageningen Bioveterinary Research
EPI_ISL_332432	The Netherlands	A/Guineafowl/Netherlands/17017775-020-024/2017	H5N6	15/12/2017	Wageningen Bioveterinary Research
EP_ISL_332430	The Netherlands	A/Chicken/Netherlands/18003041-001-005/2018	H5N6	25/05/2018	Wageningen Bioveterinary Research
EPI_ISL_332436	The Netherlands	A/Peacock/Netherlands/18000887-006/2018	H5N6	20/01/2018	Wageningen Bioveterinary Research
EPI_ISL_332437	The Netherlands	A/Turkey/Netherlands/18000887-007-010/2018	H5N6	20/01/2018	Wageningen Bioveterinary Research
EPI_ISL_292225	England	A/canada goose/England/AV58_18OPpoolEP1/2018	H5N6	05/01/2018	Animal and Plant Health Agency
EPI_ISL_292224	England	A/porchard duck/England/AVP_18_003254/2018	H5N6	10/01/2018	Animal and Plant Health Agency
EPI_ISL_292223	England	A/mute swan/England/AVP_18_001986/2017	H5N6	31/12/2017	Animal and Plant Health Agency
EPI_ISL_288412	The Netherlands	A/Tufted Duck/Netherlands/17017367-007/2017	H5N6	09/12/2017	Wageningen Bioveterinary Research
EPI_ISL_332438	The Netherlands	A/Mute swan/Netherlands/17017903-002/2017	H5N6	19/12/2017	Wageningen Bioveterinary Research
EPI_ISL_288409	The Netherlands	A/Mute Swan/Netherlands/17017367-012/2017	H5N6	09/12/2017	Wageningen Bioveterinary Research
EPI_ISL_288410	The Netherlands	A/Mute Swan/Netherlands/17017377-001/2017	H5N6	09/12/2017	Wageningen Bioveterinary Research
EPI_ISL_289714	The Netherlands	A/Black-headed Gull/Netherlands/29/2017	H5N6	18/12/2017	Erasmus Medical Center
EPI_ISL_332440	The Netherlands	A/Buzzard/Netherlands/18004242-002/2018	H5N6	15/03/2018	Wageningen Bioveterinary Research
EPI_ISL_287906	The Netherlands	A/Duck/Netherlands/17017236-001-005/2017	H5N6	07/12/2017	Wageningen Bioveterinary Research
EPI_ISL_287907	The Netherlands	A/Duck/Netherlands/17017237-001-005/2017	H5N6	07/12/2017	Wageningen Bioveterinary Research
EPI_ISL_332441	The Netherlands	A/Mallard/Netherlands/18012508-017/2018	H5N6	24/08/2018	Wageningen Bioveterinary Research
EPI_ISL_332435	The Netherlands	A/Chicken/Netherlands/18000887-005/2018	H5N6	20/01/2018	Wageningen Bioveterinary Research
EPI_ISL_302826	The Netherlands	A/Chicken/Netherlands/EMC-14/2018	H5N6	26/02/2018	Erasmus Medical Center
EPI_ISL_302825	The Netherlands	A/Chicken/Netherlands/EMC-1/2018	H5N6	26/02/2018	Erasmus Medical Center
EPI_ISL_305416	The Netherlands	A/Domestic Duck/Netherlands/EMC-2/2018	H5N6	13/03/2018	Erasmus Medical Center
EPI_ISL_305417	The Netherlands	A/Domestic Duck/Netherlands/EMC-6/2018	H5N6	13/03/2018	Erasmus Medical Center
EPI_ISL_332439	The Netherlands	A/Duck/Netherlands/18003885-001-005/2018	H5N6	13/03/2018	Wageningen Bioveterinary Research
EPI_ISL_332439	The Netherlands	A/Peregrine falcon/Netherlands/18003274-001/2018	H5N6	01/03/2018	Wageningen Bioveterinary Research
EPI_ISL_305453	Germany	A/chicken/Germany-SH/AR163-L02542/2018	H5N6	19/03/2018	Friedrich-Loeffl er-Institut
EPI_ISL_305454	Germany	A/chicken/Germany-SH/AR164-L02543/2018	H5N6	19/03/2019	Friedrich-Loeffl er-Institut
EPI_ISL_306989	Germany	A/turkey/Germany-SH/AR185-L02549/2018	H5N6	19/03/2018	Friedrich-Loeffl er-Institut

EPI_ISL_31 3226	Germany	A/white stork/Germany-NI/AR251/2018	H5N6	04/2018	Friedrich-Loeffl er-Institut
EPI_ISL_31 3227	Germany	A/buzzard/Germany-NRW/AR279/2018	H5N6	05/2018	Friedrich-Loeffl er-Institut
EPI_ISL_32 2179	Germany	A/domestic duck/Germany-MV/AR163-L02727/2018	H5N6	31/08/2018	Friedrich-Loeffl er-Institut
EPI_ISL_29 1109	Germany	A/common_porchard/Germany-BY/AR09-L0241/2017	H5N6	28/12/2017	Friedrich-Loeffl er-Institut
EPI_ISL_30 3520	Republic of Georgia	A/Mallard/Republic of Georgia/1/2018	H5N6	28/01/2018	Erasmus Medical Center
EPI_ISL_30 2824	The Netherland	A/Eurasian wigeon/Netherlands/1/2018	H5N6	07/02/2018	Erasmus Medical Center
EPI_ISL_26 8669	The Netherlands	A/T_Dk/NL Monnickendam/16013865-006-008/2016	H5N8	08/11/2016	Wageningen Bioveterinary Research
EPI_ISL_26 8630	The Netherlands	A/Dk/NL Biddinghuizen/16015083-016-020/2016	H5N8	30/11/2016	Wageningen Bioveterinary Research
EPI_ISL_30 0747	Poland	A/mute_swan/Poland/137/2017	H5N8	21/02/2017	National Veterinary Research Institute Poland, PIWet-PIB
MW026125-MW026132	Denmark	A/tufted duck/Denmark/17740-1p1/2016	H5N8	07/11/2016	Statens Serum Institut
MW026069-MW026076	Denmark	A/common buzzard/Denmark/4079-1/2017	H5N8	23/03/2017	Statens Serum Institut
EPI_ISL_28 8362	Greece	A/chicken/Greece/39_2017/2017	H5N6	06/02/2017	Animal and Plant Health Agency
EPI_ISL_28 8363	Greece	A/chicken/Greece/39_2017a/2017	H5N6	06/02/2017	Animal and Plant Health Agency
EPI_ISL_28 8364	Greece	A/chicken/Greece/39_2017b/2017	H5N6	06/02/2017	Animal and Plant Health Agency
EPI_ISL_26 2055	Germany	A/Eurasian wigeon/Germany-NI/AR249-L02143/2017	H5N8	04/01/2017	Friedrich-Loeffl er-Institut
EPI_ISL_28 2143	Italy	A/goose/Italy/17VIR6358-3/2017	H5N8	05/08/2017	Instituto Zooprofilattico Sperimentale Delle Venezie
EPI_ISL_25 5182	Italy	A/turkey/Italy/17VIR538-1/2017	H5N8	20/01/2017	Instituto Zooprofilattico Sperimentale Delle Venezie
EPI_ISL_30 0744	Poland	A/swan/Poland/32/2017	H5N8	19/01/2017	National Veterinary Research Institute Poland, PIWet-PIB
EPI_ISL_26 2058	Germany	A/greylag goose/Germany-NI/AR1395/L02144/2017	H5N8	02/02/2017	Friedrich-Loeffl er-Institut
EPI_ISL_24 3288	The Netherlands	A/mallard duck/Netherlands/32/2011	H5N2	20/10/2011	Center for Research on Influenza Pathogenesis
EPI_ISL_24 3513	The Netherlands	A/Eurasian teal/Netherlands/1/2011	H3N8	15/10/2011	Center for Research on Influenza Pathogenesis
EPI_ISL_37 3081	The Netherlands	A/Mallard/Netherlands/21/2013	H3N2	31/12/2013	Center for Research on Influenza Pathogenesis
EPI_ISL_26 7224	The Netherlands	A/mallard duck/Netherlands/16/2012	H3N2	17/08/2012	Center for Research on Influenza Pathogenesis
EPI_ISL_26 7376	The Netherlands	A/mallard duck/Netherlands/18/2012	H4N2	21/08/2012	Center for Research on Influenza Pathogenesis
EPI_ISL_24 3608	The Netherlands	A/mallard duck/Netherlands/15/2011	H6N8	14/09/2011	Center for Research on Influenza Pathogenesis
EPI_ISL_18 89692	Republic of Georgia	A/mallard/Republic of Georgia/12/2011	H10N4	11/02/2011	Erasmus Medical College
EPI_ISL_20 9380	Ukraine	A/shelduck/Yasnopolyanske/4-08-02/2011	H1N1	08/02/2011	National Scientific Center Institute of Experimental and Clinical Veterinary MedicineE
EPI_ISL_18 9700	Republic of Georgia	A/mallard/Republic of Georgia/13/2011	H6N2	26/11/2011	Erasmus Medical College
EPI_ISL_30 0712	Poland	A/chicken/Poland/263/2017	H5N8	04/03/2017	National Veterinary Research Institute Poland, PIWet-PIB

EPI_ISL_30 0744	Poland	A/swan/Poland/32/2017	H5N8	19/01/2017	National Veterinary Research Institute Poland, PIWet-PIB
EPI_ISL_30 2523	South Korea	A/Anas platyrhynchos/Korea/W612/2017	H5N6	13/12/2017	Chungbuk National University
EPI_ISL_30 2823	The Netherlands	A/Great black-backed gull/Netherlands/1/2017	H5N6	18/12/2017	Erasmus Medical Center
EPI_ISL_24 7725	Russia	A/chicken/Kalmykia/2643/2016	H5N8	21/11/2016	Vector State Research Center of Virology and Biotechnology
EPI_ISL_24 7725	Russia	A/gadwall/Kurgan/2442/2016	H5N8	27/08/2016	Vector State Research Center of Virology and Biotechnology
EPI_ISL_30 9197	Belgium	A/Brahma chicken/Belgium/6153/2017	H5N8	15/06/2017	Veterinary and Agrochemical Research Center
EPI_ISL_30 9198	Belgium	A/Guinea fowl/Belgium/6102/2017	H5N8	14/06/2017	Veterinary and Agrochemical Research Center
EPI_ISL_27 5288	Russia	A/chicken/Tatarstan/88/2017	H5N8	08/05/2017	Vector State Research Center of Virology and Biotechnology
EPI_ISL_27 5433	Russia	A/unknown/Tatarstan/86/2017	H5N8	08/05/2017	Vector State Research Center of Virology and Biotechnology
EPI_ISL_27 5432	Russia	A/unknown/Tatarstan/94/2017	H5N8	08/05/2017	Vector State Research Center of Virology and Biotechnology
EPI_ISL_28 2143	Italy	A/goose/Italy/17VIR6358-3/2017	H5N8	05/08/2017	Instituto Zooprofilattico Sperimentale Delle Venezie
EPI_ISL_27 3847	Italy	A/turkey/Italy/17VIR5878-3/2017	H5N8	23/07/2017	Instituto Zooprofilattico Sperimentale Delle Venezie
EPI_ISL_30 8782	Japan	A/jungle crow/Hyogo/2803E011/2018	H5N8	03/03/2018	Avian Zoonosis Research Center
EPI_ISL_28 9173	Japan	A/mute swan/Shimane/3211A001/2017	H5N8	05/11/2017	Avian Zoonosis Research Cente
EPI_ISL_26 8630	The Netherlands	A/T_Dk/NL Biddinghuizen/16015083-016-020/2016	H5N8	30/11/2016	Wageningen Bioveterinary Research
MW026077-MW026084	Denmark	A/common buzzard/Denmark/3794-1/2017	H5N8	03/03/2017	Statens Serum Institut
EPI_ISL_26 8669	The Netherlands	A/T_Dk/NL Monnickendam/16013865/006/008/2016	H5N8	08/11/2016	Wageningen Bioveterinary Research
MW026109-MW026116	Denmark	A/great black-backed gull/Denmark/19069-1/2016	H5N8	16/11/2016	Statens Serum Institut
MW026101-MW026107	Denmark	A/goose/Denmark/1365-1c/2017	H5N8	06/02/2017	Statens Serum Institut
EPI_ISL_30 0747	Poland	A/mute swan/Poland/137/2017	H5N8	21/02/2017	National Veterinary Research Institut Poland, PIWet-PIB
MW026085-MW026092	Denmark	A/common gull/Denmark/18577-1/2016	H5N8	16/11/2016	Statens Serum Institut
MW026117-MW026124	Denmark	A/mute swan/Denmark/19192-1/2016	H5N8	19/11/2016	Statens Serum Institut
MW026093-MW026100	Denmark	A/duck/Denmark/19062-5Sp1c/2016	H5N8	19/11/2016	Statens Serum Institut
EPI_ISL_25 5182	Italy	A/turkey/Italy/17VIR538-1/2017	H5N8	20/01/2017	Instituto Zooprofilattico Sperimentale Delle Venezie
EPI_ISL_24 0677	Russia	A/domestic duck/Siberia/103/2016	H5N8	04/10/2016	Research Institute of Experimental and Clinical Medicine
EPI_ISL_26 7359	The Netherlands	A/mallard duck/Netherlands/2/2004	H6N8	27/09/2004	Center for Research on Influenza Pathogenesis

EPI_ISL_292418	South Korea	A/mandarin duck/Korea/H69/2017	H5N6	19/12/2017	Animal and Plant Health Agency
EPI_ISL_287800	Taiwan	A/spoonbill/Taiwan/DB645/2017	H5N6	01/12/2017	Animal Health Research Institute
EPI_ISL_328973	Republic of Georgia	A/mallard duck/Georgia/3/2016	H4N6	30/09/2016	Center for Research on Influenza Pathogenesis
EPI_ISL_243664	The Netherlands	A/mallard duck/Netherlands/19/2012	H4N6	07/09/2012	Center for Research on Influenza Pathogenesis
EPI_ISL_292362	Croatia	A/teal/Croatia/86-25/2012	H4N6	02/09/2012	Croatian Veterinary Institute
EPI_ISL_132840	Pakistan	A/Khaki Campbell duck/Karachi/NARC-23963/2010	H4N6	09/12/2010	National Reference Laboratory for Poultry Diseases
EPI_ISL_387986	Bangladesh	A/duck/Bangladesh/36395/2018	H4N6	09/12/2018	St. Jude Center of Excellence for Influenza Research and Surveillance
EPI_ISL_209115	Mongolia	A/duck/Mongolia/127/2015	H4N6	26/08/2015	Jun Hatamachi Hokkaido University
EPI_ISL_231650	Russia	A/teal/Chany Lake/106_cloaca/2012	H4N6	26/08/2012	Research Institute of Experimental and Clinical Medicine
EPI_ISL_206812	China	A/chicken/Hunan/S1267/2010	H4N6	03/03/2010	State Key Laboratory of Veterinary Biotechonology
EPI_ISL_376082	China	A/duck/Yunnan/YN-1/2011	H4N6	17/12/2009	Academy of Military Medical Sciences
EPI_ISL_129199	India	A/chicken/India/WB-NIV101018/2009	H4N6	17/12/2009	National Institute of Virology
EPI_ISL_121436	Vietnam	A/duck/Vietnam/OIE-0155/2012	H4N6	20/02/2012	Jun Hatamachi Hokkaido University
EPI_ISL_376208	The Netherlands	A/mallard/Netherlands/89/2017	H4N6	26/08/2017	Center for Research on Influenza Pathogenesis
EPI_ISL_267342	The Netherlands	A/mallard duck/Netherlands/2/2013	H3N6	10/09/2013	Center for Research on Influenza Pathogenesis
EPI_ISL_158422	Sweden	A/mallard/Sweden/104690/2009	H4N6	21/11/2009	Linnaeus University
EPI_ISL_158416	Sweden	A/mallard/Sweden/105137/2009	H4N6	27/11/2009	Linnaeus University
EPI_ISL_89981	Czech Republic	A/mallard/Czech Republic/13579-84K/2010	H4N6	13/09/2010	National Reference Laboratory for Avian Influenza and Newcastle Disease
MT755876	Denmark	A/mallard/Denmark/13996-26p2w/2017	H4N6	11/09/2017	Statens Serum Institut
MT755875	Denmark	A/mallard/Denmark/13996-27p2w/2017	H4N6	11/09/2017	Statens Serum Institut
MT755877	Denmark	A/mallard/Denmark/13997-29p2w/2017	H4N6	11/09/2017	Statens Serum Institut
MT755878 MT755879	Denmark	A/mallard/Denmark/19753-36p1w/2016	H4N6	28/11/2016	Statens Serum Institut
MT755880	Denmark	A/mallard/Denmark/14716-38p2w/2018	H4N6	22/10/2018	Statens Serum Institut
EPI_ISL_373090	The Netherlands	A/mallard/Netherlands/39/2015	H4N6	08/09/2015	-
EPI_ISL_158419	Sweden	A/mallard/Sweden/124418/2010	H4N6	03/11/2010	Linnaeus University
MT755881	Denmark	A/mallard/Denmark/15487-54p2w/2018	H4N6	06/11/2018	Statens Serum Institut
EPI_ISL_332402	China	A/Jiangsu/1/2018	H5N6	06/11/2018	Jiangsu Provincial Center for Disease Control and Prevention
EPI_ISL_163493	China	A/Sichuan/26221/2014	H5N6	21/04/2014	WHO Chinese National Influenza Center
EPI_ISL_150054	Sweden	A/mallard/Sweden/40/2002	H5N6	12/11/2002	Erasmus Medical Center

EPI_ISL_298640	Israel	A/turkey/Israel/1045/2016	H5N8	20/12/2016	Kimron Veterinari Institute
EPI_ISL_389751	Egypt	A/chicken/Egypt/M14081D/2017	H5N8	10/05/2018	St. Jude Center of Excellence for influenza Research and Surivellance
EPI_ISL_247720	Russia	A/chicken/Voronezh/20/2017	H5N8	06/06/2017	Vector State Research Center of Virology and Biotechnology
EPI_ISL_247722	Russia	A/goose/Krasnodar/3144/2017	H5N8	06/01/2017	Vector State Research Center of Virology and Biotechnology
EPI_ISL_266537	Belgium	A/peacock/Belgium/1017/2017	H5N8	08/02/2017	Veterinary and Agrochemical Research Center
EPI_ISL_266418	South Korea	A/common teal/Korea/W549/2016	H5N8	15/12/2016	Chungbuk National University
EPI_ISL_287565	The Netherlands	A/M_swan/NL Groningen/16015826-001/2016	H5N5	13/12/2015	Wageningen Bioveterinary Research
